# The regulation of the homeostasis and regeneration of peripheral nerve is distinct from the CNS and independent of a stem cell population

**DOI:** 10.1242/dev.170316

**Published:** 2018-12-14

**Authors:** Salome Stierli, Ilaria Napoli, Ian J. White, Anne-Laure Cattin, Anthony Monteza Cabrejos, Noelia Garcia Calavia, Liza Malong, Sara Ribeiro, Julie Nihouarn, Richard Williams, Kaylene M. Young, William D. Richardson, Alison C. Lloyd

**Affiliations:** 1 MRC Laboratory for Molecular Cell Biology, University College London, Gower Street, London WC1E 6BT, UK; 2 Menzies Institute for Medical Research, University of Tasmania, Hobart, TAS 7000, Australia; 3 Wolfson Institute for Biomedical Research, University College London (UCL), Gower Street, London WC1E 6BT, UK; 4 UCL Cancer Institute, University College London, Gower Street, London WC1E 6BT, UK

**Keywords:** Tissue homeostasis, Tissue regeneration, Stem cells, PNS, CNS, Schwann cells

## Abstract

Peripheral nerves are highly regenerative, in contrast to the poor regenerative capabilities of the central nervous system (CNS). Here, we show that adult peripheral nerve is a more quiescent tissue than the CNS, yet all cell types within a peripheral nerve proliferate efficiently following injury. Moreover, whereas oligodendrocytes are produced throughout life from a precursor pool, we find that the corresponding cell of the peripheral nervous system, the myelinating Schwann cell (mSC), does not turn over in the adult. However, following injury, all mSCs can dedifferentiate to the proliferating progenitor-like Schwann cells (SCs) that orchestrate the regenerative response. Lineage analysis shows that these newly migratory, progenitor-like cells redifferentiate to form new tissue at the injury site and maintain their lineage, but can switch to become a non-myelinating SC. In contrast, increased plasticity is observed during tumourigenesis. These findings show that peripheral nerves have a distinct mechanism for maintaining homeostasis and can regenerate without the need for an additional stem cell population.

This article has an associated ‘The people behind the papers’ interview.

## Abstract (precis)

**Highlighted Article:** Lineage analysis and long-term labelling studies show that peripheral nerve regeneration is underpinned by the proliferative plasticity of mature cells rather than the activation of a stem cell population.

## INTRODUCTION

Once formed in the adult, peripheral nerves are relatively stable structures, befitting their role in transmitting signals back and forth between tissues and organs and the central nervous system (CNS). However, in contrast to the CNS, peripheral nerves are able to regenerate following an injury (Mahar and Cavalli, 2018). This requires not only the regrowth of the neurons but the creation of new tissue to repair the wound site, together with the remodelling of the remaining nerve tissue to provide an environment conducive for axonal regrowth (Cattin and Lloyd, 2016; Zochodne, 2012). Crucial to this regenerative process is the main glial cell of the peripheral nervous system (PNS), the Schwann cell (SC). In the adult, SCs exist in one of two states: myelinating Schwann cells (mSCs), which myelinate larger axons in a 1:1 ratio, and non-myelinating Schwann cells (nmSCs), which bundle together groups of smaller axons in structures known as Remak bundles (Harty and Monk, 2017; Monk et al., 2015). Following injury, these highly specialised cells have the capacity to dedifferentiate to a proliferating, progenitor-like SC, which orchestrates the regenerative response (Jessen and Mirsky, 2016; Napoli et al., 2012). The roles of SCs in this process include guiding regrowing axons across the injury site (Cattin et al., 2015; Parrinello et al., 2010), opening the blood-nerve barrier (BNB) and controlling the inflammatory response (Napoli et al., 2012), remodelling the nerve environment (Brosius Lutz et al., 2017) and promoting the growth and survival of the regrowing axons (Arthur-Farraj et al., 2012; Fontana et al., 2012). Once the axons have regrown, the dedifferentiated SCs are thought to redifferentiate in response to axonal signals, and the regenerative state resolves to return to the homeostatic state (Cattin and Lloyd, 2016; Zochodne, 2008).

The behaviour of mSCs is in stark contrast to that of the comparable cells of the CNS, the oligodendrocyte (OLs). Once an OL has myelinated a neuron, it is postmitotic in that it cannot return to a proliferative state. Moreover, new OLs are produced throughout life, either to replace OLs or to myelinate previously unmyelinated axon segments (Birey et al., 2017; Young et al., 2013). This *de novo* myelination in adulthood is thought to contribute to the plasticity of the brain in processes such as learning and memory (Kaller et al., 2017; McKenzie et al., 2014). New OLs are produced from a continuously slowly proliferating pool of progenitor cells that exist throughout the CNS, known as oligodendrocyte progenitor cells (OPCs) (Dimou and Simons, 2017; Kang et al., 2010). These cells continuously produce new OLs in the adult (Young et al., 2013) and following demyelination events in pathologies such as multiple sclerosis (Domingues et al., 2016).

It is not clear why the CNS and PNS have evolved distinct mechanisms to produce new cells and have such different regenerative capabilities. Moreover, the apparent lack of a stem cell/progenitor population in the PNS to produce new cells, either during homeostasis or following injury, is unusual for a mammalian tissue. This has led to speculation that an additional stem cell population contributes to the production of new SCs during the regenerative process (Amoh et al., 2005; Chen et al., 2012; McKenzie et al., 2006), and that SCs retain some of the multipotency that SC precursors exhibit during development in order to regenerate new nerve tissue (Petersen and Adameyko, 2017). In this study, we have characterised the behaviour of all cell types within peripheral nerve during homeostasis and during the regenerative process. Moreover, we have used lineage analysis to track the behaviour and fate of mSCs. We find that peripheral nerve is a highly quiescent tissue and that, in contrast to OLs, mSCs do not turn over in adulthood. Following injury, however, all cell types within the nerve proliferate, with close to 100% of mSCs entering the cell cycle to become migratory, progenitor-like SCs, which orchestrate the multicellular nerve regeneration process without the requirement for a distinct SC stem cell population. Lineage analysis shows that these ‘repair’ SCs retain the SC lineage, but can switch from a mSC to a nmSC. In contrast, we find that this restriction breaks down during SC tumourigenesis, when these cells show increased plasticity. This work shows that peripheral nerve is a tissue with a distinct mechanism for both maintaining homeostasis and regenerating following injury – in that cells rarely turn over in the homeostatic state, whereas all cells in the tissue proliferate and contribute to the repair of the damaged nerve. This study also demonstrates the remarkable stability of glia in the PNS, despite retaining the ability to efficiently convert to a progenitor-like SC following injury, providing a further illustration of the diversity of stem/progenitor cell phenotypes that exist in mammalian tissues.

## RESULTS

### Identification of the cell composition of peripheral nerve

**Fig. 1.**
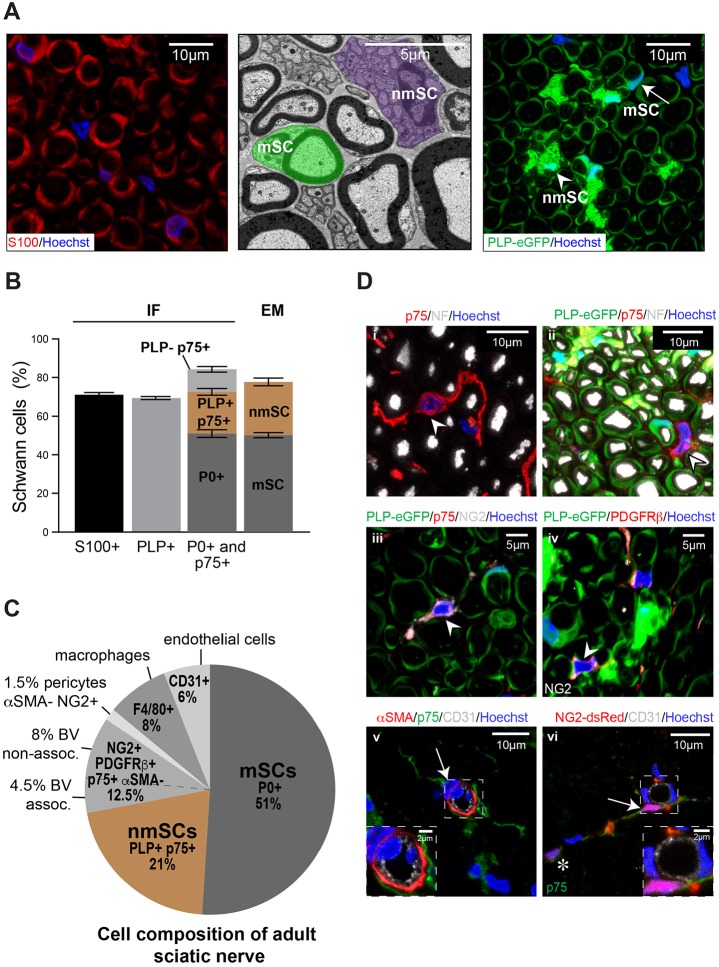
**Identification of the cell composition of peripheral nerve.** (A) Representative confocal IF and EM images, showing the SC population in transverse sections of mouse sciatic nerve. SCs are labelled by staining with anti-S100 antibody (red) or endogenously in *PLP-eGFP* mice (green). In the EM image, a nmSC is coloured purple while a typical mSC is coloured green. (B) Quantification shows the proportions of SCs in mouse sciatic nerve (*n*=4 mice, mean±s.e.m.). (C) Pie chart showing the percentages of individual cell types within sciatic nerve. Cryosections of sciatic nerves were immunolabelled with cell type-specific markers as indicated (*n*=4). The NG2^+^/PDGFRβ^+^/p75^+^/αSMA^−^ population is classified as either associated or non-associated with CD31^+^ blood vessels (BV). Pericytes are defined as NG2^+^/PDGFRβ^+^/αSMA^+^. (D) Representative images of transverse mouse sciatic nerve sections labelled as indicated. (i) Arrowhead indicates a p75^+^ cell (red) that is not associated with axons [labelled with antibody against neurofilament (NF), white]. (ii) Arrowhead indicates a p75^+^ cell (red) that is not associated with axons (labelled for NF, white) and does not express eGFP in nerves isolated from *PLP-eGFP* mice. (iii) Arrowhead indicates a PLP-eGFP^−^/p75^+^/NG2^+^ cell. (iv) Arrowhead indicates a PLP-eGFP^−^/NG2^+^/PDGFRβ^+^ cell. (v) Arrow indicates an αSMA^+^/p75^−^ pericyte in close contact with a CD31^+^ blood vessel (white). (vi) Arrow indicates a p75^+^ cell that co-expresses dsRed in nerves isolated from *NG2-dsRed* mice and is loosely associated with a CD31^+^ blood vessel. The asterisk indicates a p75^+^/dsRed^+^ cell that is not associated with a blood vessel. (v,vi) Dashed line boxes indicate the regions that are shown at higher magnification in insets. See also Fig. S1.

In order to determine the composition and turnover of cells found in a peripheral nerve, we initially systematically determined the cell composition within the endoneurium of mouse sciatic nerve. To do this, we used a number of transgenic mice with lineage-specific expression of fluorescent labels, along with immunostaining of endogenous markers to quantify the prevalence of each cell type using immunofluorescence (IF) and electron microscopy (EM) analysis. Consistent with previous findings (Salonen et al., 1988), we found that the vast majority of cells within the sciatic nerve are SCs (∼70%), as determined by staining for the cytoplasmic SC marker S100 (S100B) and by EM analysis (Fig. 1A,B). Moreover, these results were confirmed by imaging nerve sections from a transgenic mouse in which all SCs express eGFP (*PLP-eGFP* mice) (Fig. 1A) (Mallon et al., 2002), and by immunostaining for myelin protein zero (P0; Mpz) (Fig. S1A) and p75 (Ngfr) (Fig. S1B). This analysis also confirmed that the majority of cells were mSCs (51%), with the ratio of mSCs to nmSCs (21%) roughly 2:1 (Fig. 1B,C, Fig. S1C).

p75 has been used extensively as a marker to label both nmSCs and the dedifferentiated SCs that form following an injury (Jessen et al., 2015). However, we also found a significant proportion of p75^+^ cells that did not appear to be associated with axons and had a distinct elongated morphology (Fig. 1D). Moreover, these p75^+^ cells did not express eGFP in nerves isolated from *PLP-eGFP* mice (Fig. 1D) or tdTomato in nerves from *P0-Cre:tdTomato* mice that express tdTomato in all SCs (Fig. S1D). Instead, we found that these cells (p75^+^/eGFP^−^) expressed the commonly used markers for pericytes, NG2 (Cspg4) and PDGFRβ (Fig. 1D) (Armulik et al., 2011). Moreover, we confirmed NG2 expression in these cells, by analysing nerves from mice that express dsRed by virtue of an NG2-driven expression construct (*NG2-dsRed mice*) (Zhu et al., 2008), and found a complete overlap between NG2/PDGFRβ immunostaining and dsRed expression (Fig. 1D, Fig. S1E).

We could distinguish three populations of NG2^+^/PDGFRβ^+^ cells within the nerve endoneurium. The first population (1.5% of total cells within the endoneurium) had all the characteristics of classical pericytes. They co-expressed NG2 and PDGFRβ (data not shown), were negative for p75 and expressed α-smooth muscle actin (αSMA; Acta2) (Fig. 1D). Moreover, they were found tightly associated around blood vessels [CD31^+^ (Pecam1^+^) cells] (Fig. 1D), consistent with a pericyte population that could be observed within the basal lamina around the blood vessels by EM (Fig. S1F). A much larger population of NG2^+^/PDGFRβ^+^ cells (12.5%) was p75^+^ (Fig. 1C). They were also S100^–^ (Fig. S1G) and were not labelled in *PLP-eGFP* mice or in *P0-Cre:tdTomato* mice (Fig. 1D, Fig. S1D), and so appeared not to be SCs or derived from SCs. We also noticed that a proportion of these cells were found loosely associated with blood vessels (4.5%) (Fig. 1D), whereas others appeared to be within the endoneurium away from blood vessels (Fig. 1D, Fig. S1E); thus, although we were unable to distinguish these two populations by markers, we classified them as blood vessel associated (4.5%) or non-associated (8%) (Fig. 1C, Fig. S1C). It is important to note that EM analysis showed that, while some of these cells were associated with blood vessels, they were not found within the basal lamina of the vessels (Fig. S1H). EM analysis also showed high amounts of endoplasmic reticulum within these cells, indicating that they are cells that have provisionally been identified as neural crest-derived fibroblasts by others (Joseph et al., 2004) (Fig. S1H).

The remaining cells of peripheral nerve were shown to be endothelial cells (6%, CD31^+^) and macrophages [8%, F4/80^+^ (Adgre1^+^)/Iba1^+^] (Fig. 1C, Fig. S1C). We did not detect other inflammatory cells, such as mast cells, neutrophils or dendritic cells, within the uninjured nerves (data not shown). In conclusion, we have been able to identify all cell types within the endoneurium of the nerve and have characterised the tools to enable us to trace their behaviour in normal nerve and following injury.

### mSCs do not proliferate in the adult

**Fig. 2.**
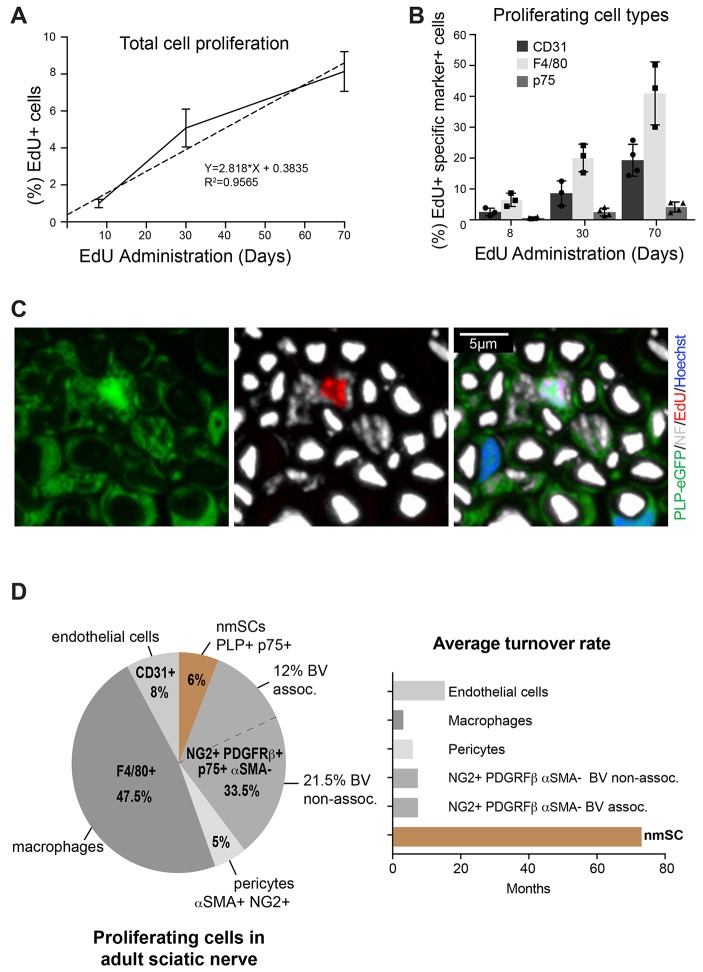
**Peripheral nerve is a highly quiescent tissue.** (A) EdU was administered continuously in the drinking water of wild-type mice for 8, 30 and 70 days. Cryosections of sciatic nerve were processed to detect EdU. Graph shows the mean percentages (±s.e.m.) of EdU^+^ cells that accumulate over time (*n*=3-4 mice). (B) Cryosections of sciatic nerves were labelled with cell-specific markers and processed to detect EdU^+^ cells (*n*=3-4 mice, ±s.e.m.). (C) 3D projection of a confocal image of a 20 μm cryosection of sciatic nerve isolated from a *PLP-eGFP* mouse treated with EdU continuously for 30 days, showing an EdU^+^ (red) eGFP^+^ nmSC associated with small axons [labelled with antibody against neurofilament (NF), white]. (D) Pie chart showing the proportion of proliferating cell types in adult sciatic nerve in mice treated with EdU for 30 days (*n*=6 mice). The NG2^+^/PDGFRβ^+^/p75^+^/αSMA^−^ population is classified as either associated or non-associated with CD31^+^ blood vessels (BV). Graph shows the calculated turnover time for each cell population. See also Fig. S2 and Movie 1.

The CNS is relatively quiescent compared with many tissues of the body; however, cumulative long-term 5-ethynyl-2′-deoxyuridine (EdU) labelling studies have shown that essentially all OPCs proliferate, albeit slowly, with turnover times ranging from ∼1 week to >1 month in different regions of the brain, spinal cord and optic nerve (Young et al., 2013). To determine the overall proliferation rate of cells within the endoneurium of peripheral sciatic nerve, we used the same protocol of cumulative EdU labelling to mark proliferating cells in adult mice. For each time point (8, 30 and 70 days), we calculated the percentage of cells that had accumulated EdU and this increased in a linear manner over time, showing that the proliferation rate remained constant during early adulthood (Fig. 2A). However, the overall proliferation rate was very low (∼0.8% per week), with fewer than 10% of the cells labelled within the 70-day time period. This implies that if all cells within the nerve were proliferating at the same rate, the tissue turnover time would be ∼2.4 years. These findings highlight the quiescent nature of peripheral nerve.

To determine the identity of the proliferating cells within the nerve, we used the cell type-specific markers defined above (Fig. 2B, Fig. S2A). Analysis of the long-term labelling studies showed that mSCs appeared to be completely quiescent, as we failed to detect a single EdU^+^ mSC over the 70-day labelling period. This implies that mSCs, once formed, do not turn over. In contrast, we detected a very low level of EdU^+^ nmSCs in Remak bundles (Fig. 2B). We confirmed the identity of these cells by the detection of EdU^+^/eGFP^+^ cells in EdU-dosed *PLP-eGFP* mice (Fig. 2C, Fig. S2B) and the association of EdU^+^ nuclei within eGFP^+^ Remak structures in 3D reconstructions (Movie 1). However, we found that the nmSC turnover rate was extremely slow (∼72 months) (Fig. 2D). Thus, the glial cells of the PNS show very different proliferative dynamics to the corresponding cells of the CNS. No new mSCs, and very few new nmSCs, are produced in the adult, whereas new OLs are produced throughout life (Young et al., 2013).

The most highly proliferative cell type in peripheral nerve was the resident macrophages, with a turnover time of ∼4 months (Fig. 2D, Fig. S2A). This is somewhat similar to the turnover rate of the resident macrophage population (microglia) in the brain (Askew et al., 2017; Tay et al., 2017). All other cell types also showed low, but detectable, levels of turnover, with endothelial cells, NG2^+^/PDGFRβ^+^/p75^+^/αSMA^−^ cells and pericytes showing approximate turnover rates of 15, 7.5 and 6 months, respectively (Fig. 2D, Fig. S2A).

### All mSCs proliferate following injury

The analysis of homeostatic peripheral nerve demonstrated a mostly quiescent tissue, consistent with its stable architecture. However, peripheral nerves are highly regenerative, requiring that the tissue retains plasticity. Following an injury, the distal stump of the nerve undergoes extensive breakdown, followed by clearance of debris, regrowth and remodelling to reform a functional nerve (Cattin and Lloyd, 2016; Zochodne, 2008). SCs have a key role in orchestrating the regenerative process, and have been reported to dedifferentiate following injury to pursue this role (Cattin and Lloyd, 2016; Jessen and Mirsky, 2016). However, it remains unclear whether all SCs have this capacity, whether a smaller ‘stem-like’ compartment is involved or whether new SCs derive from other stem cell compartments to regenerate the nerve. To distinguish between these possibilities, we performed lineage analysis using a transgenic mouse, which allows Cre expression to be specifically induced in mSCs in the adult (*P0-CreER^T2^:YFP*) following tamoxifen administration (Leone et al., 2003; Ribeiro et al., 2013).

**Fig. 3.**
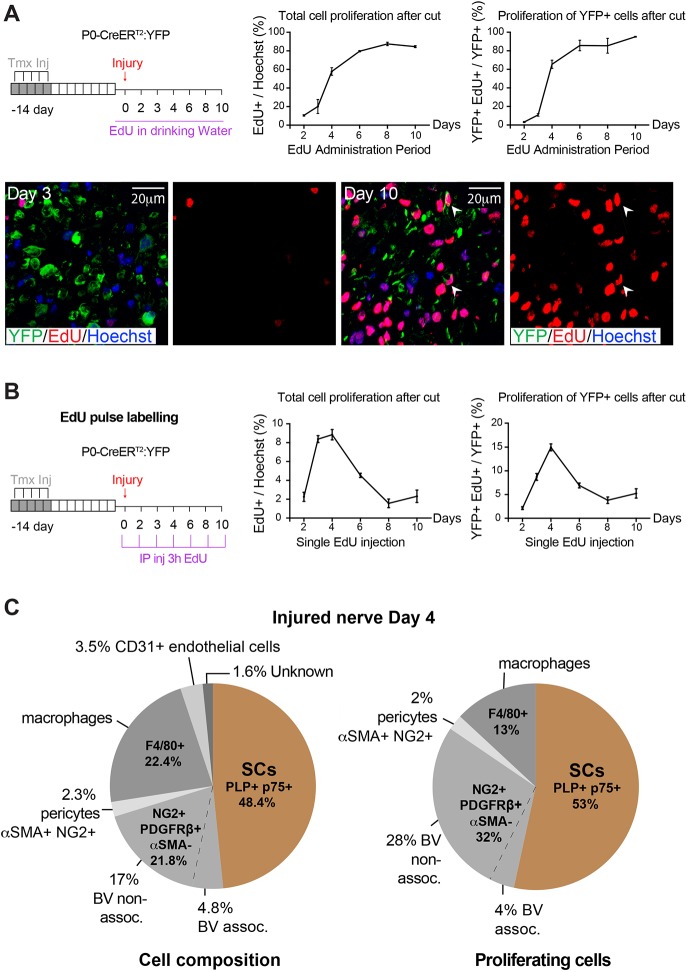
**All mSCs proliferate following injury.** (A) Schematic showing the protocol to assess accumulated proliferation of mSCs following injury. *P0-CreER^T2^:YFP* mice were treated with tamoxifen (Tmx) for 5 days to specifically label mSCs with YFP. Fourteen days later, the right sciatic nerve was transected, and EdU was administered continuously in the drinking water for 10 days. Mice were harvested at the indicated time points and proliferating (EdU^+^) cells were counted in cryosections of the distal stump (downstream of the newly formed bridge region) of sciatic nerve processed for IF staining. Graphs show the percentage of proliferating cells (EdU^+^/Hoechst^+^) or the percentage of proliferating mSCs (YFP^+^, EdU^+^/YFP^+^) plotted against the time of EdU administration (*n*=4 mice, ±s.e.m.). Representative images show that few mSCs have proliferated at Day 3 following injury. In contrast, almost all mSCs have proliferated by Day 10 postinjury. Arrowheads indicate proliferating mSCs. (B) Schematic showing the protocol used to perform a temporal analysis of the proliferation of mSCs following injury. Mice were treated as in A, but EdU was administered by IP, 3 h prior to the culling of the animals at the indicated times. Graphs show the percentages of all proliferating cells (EdU^+^/Hoechst^+^) or the percentages of proliferating mSCs (YFP^+^, EdU^+^/YFP^+^) at the indicated time points (*n*=4-6 mice, ±s.e.m.). (C) Pie charts show the cell composition and relative contribution of each cell type to the proliferating population in the distal stump of an injured sciatic nerve on Day 4 following injury, which is the peak time of proliferation (*n*=8 mice). The NG2^+^/PDGFRβ^+^/p75^+^/αSMA^−^ population is classified as either associated or non-associated with CD31^+^ blood vessels (BV). See also Fig. S3.

To analyse the initial stages of the regenerative process and to investigate the proliferative potential of mSCs, we performed cumulative EdU labelling following sciatic nerve transection in *P0-CreER^T2^:YFP* adult mice, 14 days following tamoxifen treatment (Fig. 3A). Analysis of the distal stump (downstream of the cut and the newly formed bridge region) showed a dramatic switch in the proliferative status of the nerve, with >80% of cells within the nerve accumulating EdU by Day 8 following injury (Fig. 3A). Remarkably, despite being completely quiescent in the adult nerve, >80% of the YFP^+^ (mSC-derived) cells in the nerve proliferated within 6 days of the injury, with close to 100% proliferating by Day 10. This result showed that, despite being completely quiescent in the adult, all mSCs have the capacity to proliferate following an injury (Fig. 3A, Fig. S3A).

In order to determine the kinetics of mSC proliferation following an injury, we performed EdU pulse labelling after nerve transection and found that the peak mSC proliferation was at Day 4 following injury, with the rates returning to lower, but still detectable, levels at Day 8-10 following injury (Fig. 3B, Fig. S3B). Total cell proliferation followed a similar trend, demonstrating a robust proliferative burst of all the identified cell types within the nerve, as the myelin structures break down within the injured nerve (Fig. 3B, Fig. S3C). Notably, the endothelial cells in the distal stump did not enter the cell cycle until after Day 4, indicating that, at this time point, new blood vessels have not formed within the distal stump. This is in contrast to what was reported previously for the bridge region of a transected nerve, where newly formed blood vessels have an important role in the regenerative process (Cattin et al., 2015). This analysis also showed there was an increase in the proportion of two cell types within the nerve: macrophages that increased from 8% to 22%, following the recruitment of monocytes from the blood stream, and NG2^+^, PDGFRβ^+^ αSMA^−^ cells that increased from 12% to 22%, reflecting the higher proliferative rate of this cell type (Fig. 3C, Fig. S3D). Furthermore, neutrophils and other inflammatory cells are recruited into regenerating nerve, most likely accounting for the minor unknown cell population at this time point (Napoli et al., 2012).

**Fig. 4.**
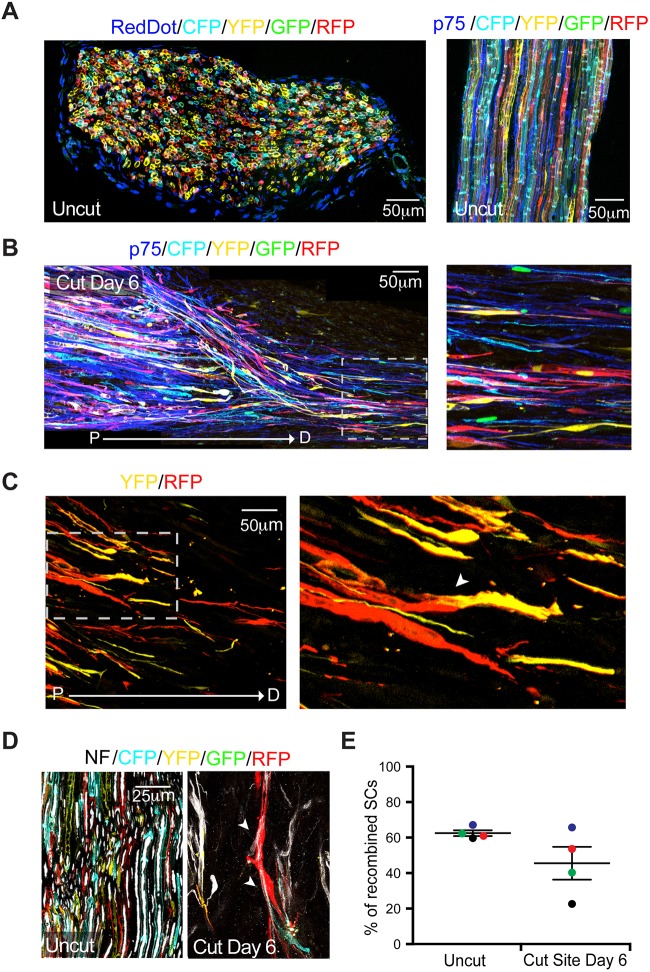
**mSCs become migratory following injury to guide regrowing axons across the injury site.** (A) Representative images of transverse (left) and longitudinal (right) cryosections of uninjured contralateral sciatic nerve, showing the high recombination rate in *P0-CreER^T2^:Confetti* mice following tamoxifen administration. Nuclei were labelled with RedDot (left), and anti-p75 antibody was used to label nmSCs (right). (B) Representative longitudinal cryosection of a transected sciatic nerve (left). The dashed line boxed area is shown at higher magnification on the right, showing that cells derived from mSCs have migrated into the nerve bridge at Day 6 following nerve injury. An antibody against p75 was used to label dedifferentiated SCs. P, proximal; D, distal. (C) Representative longitudinal cryosection of the bridge of a transected sciatic nerve (left). The dashed line boxed area is shown at higher magnification on the right. Arrowhead indicates a cord of two migrating SCs that have derived from different mSCs (YFP^+^ and RFP^+^). (D) Representative images of longitudinal sections of an uninjured and transected sciatic nerve on Day 6 following injury shows that dedifferentiated SCs derived from mSCs associate with axons while migrating into the nerve bridge. An antibody against neurofilament (NF) was used to label the axons (white). Arrowheads indicate migrating SCs associated with axons. (E) Graph shows the percentage of recombined SCs [tdTomato/(mSCs+nmSCs)] in the bridge of the injured sciatic nerve of *P0-CreER^T2^:tdTomato* mice on Day 6 after injury and in the contralateral uninjured sciatic nerve (uncut). Each coloured dot represents an individual animal. Lines show mean±s.e.m. See also Fig. S4.

A crucial aspect of SC behaviour during the regeneration of a transected nerve is that they become migratory, forming cellular cords that transport regrowing axons across the injury site (Parrinello et al., 2010). However, it is not clear whether both nmSCs and mSCs are able to undergo this marked change in migratory behaviour. To track the migration of individual SCs, we administered tamoxifen to *P0-CreER^T2^:Confetti* mice (Snippert et al., 2010), so that individual mSCs stochastically expressed a combination of four fluorophores (nuclear localised GFP, membrane-targeted Cyan and cytoplasmic RFP or YFP), generating ten possible distinct colour combinations (Fig. 4A). Following injury, SCs of each colour could be observed migrating into the bridge (Fig. 4B). Moreover, we found different coloured cells migrating next to each other in the cellular cords (Fig. 4C), and these mSC-derived cells were associated with axons in the injury region (Fig. 4D, Fig. S4A). These results show that previously quiescent mSCs can undergo extensive structural and functional remodelling to become highly migratory cells within a few days of an injury, and that they are a polyclonal population. We also observed a significant number of p75^+^ cells in the migrating cellular cords that were not labelled with a confetti-derived fluorophore (Fig. 4B). This suggested that nmSC-derived cells also contributed to the SC population migrating out of the nerve stumps into the bridge.

To confirm that the non-labelled p75^+^ population was SCs and not the NG2^+^/PDGFRβ^+^ population, we took a number of approaches. Analysis of transverse sections of the bridge region from *PLP-eGFP:NG2-dsRed* mice found that only 5.14±2.5% (mean±s.e.m., *n*=4) of the cells in the bridge were NG2^+^/p75^+^/eGFP^−^. Moreover, longitudinal sections of the bridge region showed that these cells were not present within the migrating cell cords (Fig. S4C). In addition, analysis of transverse sections of the bridge region from *P0-Cre:tdTomato* mice (in which all SCs are labelled with tdTomato) revealed that the vast majority of p75^+^ within the bridge are also tdTomato positive [95.2±2.1% (mean±s.e.m., *n*=6)]. Together, these results demonstrate that the migratory population is derived from a combination of nmSCs and mSCs. To determine the proportion of mSCs versus nmSCs, we compared the percentage of recombined SCs in the uncut contralateral nerve with that of SCs migrating in the bridge region of the injured nerve in individual *P0-CreER^T2^:tdTomato* mice (Madisen et al., 2010). More than 80% of mSCs were tdTomato^+^ in the uninjured nerve (Fig. S4B), yet in three of four mice analysed, the proportion of recombined SCs was lower in the bridge than in the contralateral uncut nerve (Fig. 4E, Fig. S4D). These results suggest that both nmSC- and mSC-derived cells migrate into the bridge following injury, but that nmSC-derived cells tend to make a greater contribution to this process.

A second role for SCs during the regenerative response is to contribute to the clearance of the axonal and myelin debris that accumulates in the distal stump, as the axons degenerate and mSCs dedifferentiate following a transection injury (Brosius Lutz et al., 2017; Fernandez-Valle et al., 1995; Gomez-Sanchez et al., 2015). Using *P0-CreER^T2^:tdTomato* mice, we could visualise mSC-derived cells that had engulfed myelin debris in the distal stump of injured nerves (Fig. S4E), consistent with previous studies suggesting that cells derived from mSCs are able to clear the debris in injured nerves (Brosius Lutz et al., 2017). In contrast, p75^+^/tdTomato^−^ cells do not appear to contain myelin debris, probably reflecting that nmSCs do not have access to the myelin debris.

### The regenerated nerve has structural abnormalities

**Fig. 5.**
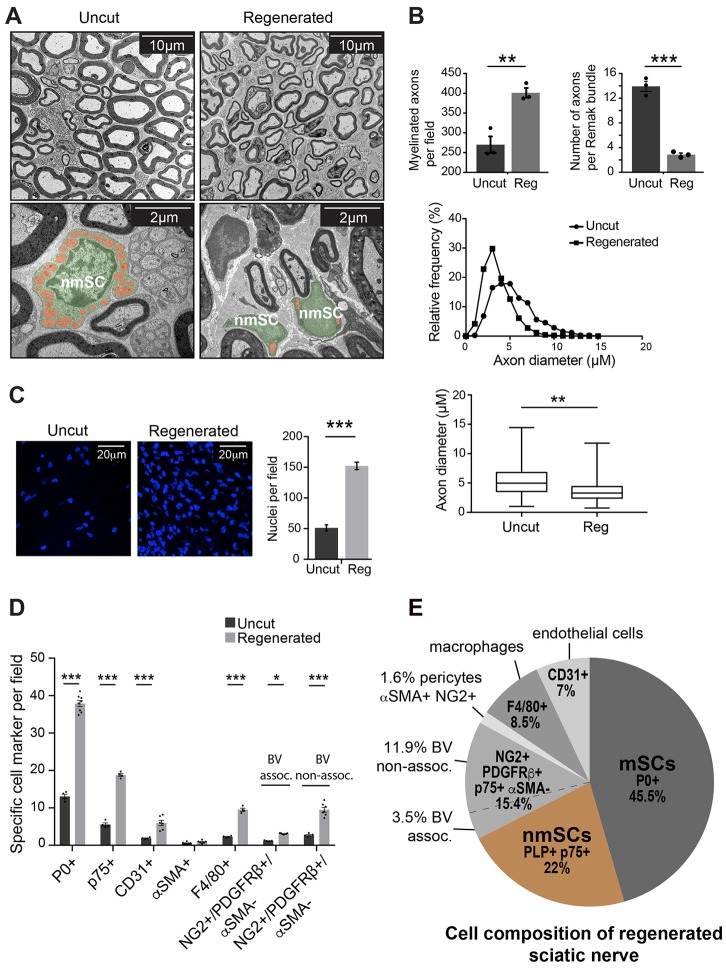
**Regenerated nerve is distinct from uninjured nerve.** (A) Representative EM images of uncut contralateral or regenerated nerve taken 6 months following injury. Note that the regenerated nerve has smaller axons and more myelinated axons per field compared with the uncut nerve. The lower panels show a higher magnification of the EM images, in which nmSCs are highlighted in green and small calibre axons in orange to show the decreased numbers of axons per Remak bundle in the regenerated nerve. (B) Quantifications of A, showing the density of myelinated axons, the number of axons per Remak bundle (*n*=3 mice, mean±s.e.m.) and the axon diameters (*n*=8 mice) in uncut and regenerated nerve. (C) Representative confocal images of transverse sections of uninjured and regenerated sciatic nerve, 6 months after injury, showing Hoechst^+^ cells (blue). Graph shows the quantification of the number of nuclei per field (*n*=8 mice, mean±s.e.m.). (D) Cryosections of regenerated and uninjured sciatic nerve were immunolabelled for cell type-specific markers as indicated, 6 months following injury, and the number of the different cell types was quantified per field (*n*=4-8 mice, mean±s.e.m., two-way ANOVA was used). (E) Pie chart shows the cell composition of regenerated sciatic nerve 6 months following injury (*n*=4-8 mice). The NG2^+^/PDGFRβ^+^/p75^+^/αSMA^−^ population is classified as either associated or non-associated with CD31^+^ blood vessels (BV). See also Fig. S5. *P<0.05, **P<0.01, ***P<0.001.

It is well established that a regenerated nerve is distinct from an uninjured nerve. In particular, there is an increase in the number of axons owing to increased axonal sprouting during the regenerative process (Bray and Aguayo, 1974; Fawcett and Keynes, 1990; Friede and Bischhausen, 1980; Gutmann and Sanders, 1943). Using EM and the cell-specific markers characterised above, we performed a detailed analysis of the structure and cell composition of the fully regenerated nerve at 6 months following injury. Consistent with previous studies (Salonen et al., 1988; Zochodne, 2008), we found an increase in the density and number of myelinated axons in regenerated nerves and a decrease in the average size of these axons (Fig. 5A,B, Fig. S5B,C). We also detected a striking difference in the structure of Remak bundles with a large decrease in the number of axons within individual Remak structures (Fig. 5A,B). The regenerated nerves also showed a more than threefold increase in cell density (Fig. 5C). This represented a proportional increase of all cell types within the nerve (Fig. 5D,E, Fig. S5A,B), indicating that interactions between the cell types act to maintain the composition of the tissue, with the increased number of nmSCs likely explaining the decreased number of axons within each Remak structure. Importantly, we were able to identify all the cells within the regenerated nerve (Fig. 5E), suggesting that all cell types retain their identity during the regeneration process.

**Fig. 6.**
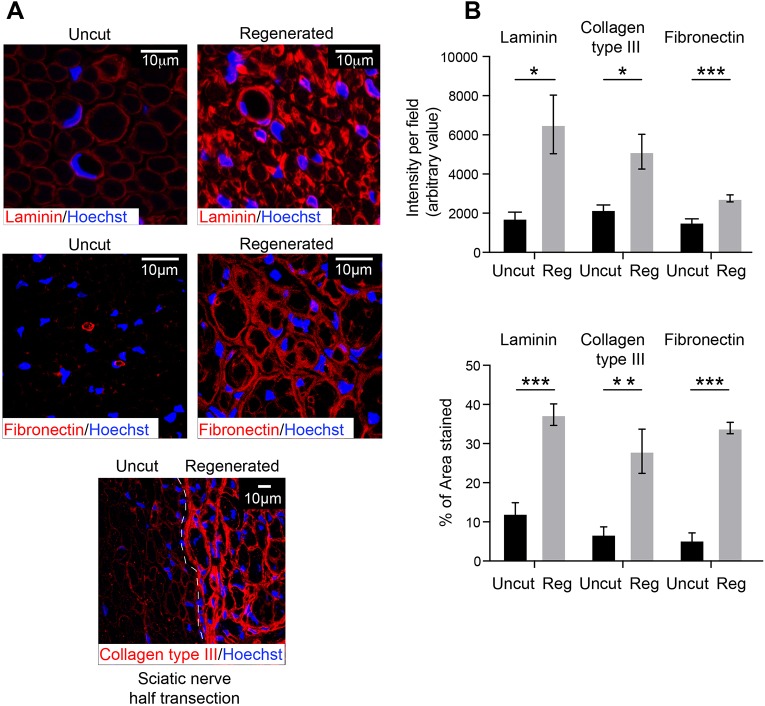
**The levels of ECM proteins remain high in regenerated nerve.** (A) Representative confocal images of transverse sections of uninjured sciatic nerve, and the distal stump of regenerated sciatic nerve, 6 months following injury, immunostained for the indicated ECM proteins. (B) Graphs show quantifications of A (*n*=6 mice, mean±s.e.m.). *P<0.05, **P<0.01, ***P<0.001.

A striking observation was that, in regenerated nerve, the level of extracellular matrix (ECM) was dramatically different compared with that in an uninjured nerve. ECM levels increase during an injury/regenerative process (Eming et al., 2017), but we found that the additional ECM was not cleared from the regenerated nerve. There were much higher levels of laminin deposition within the basal lamina around the SCs and blood vessels in regenerated nerve (Fig. 6A,B). Moreover, the levels of fibronectin and collagen III were considerably higher throughout the regenerated nerve (Fig. 6A,B). These findings are consistent with the perceived fibrotic nature of repaired tissue and suggest that, whereas many cellular changes and the inflammatory response associated with a regenerative process successfully resolve, the increased ECM deposition associated with tissue regeneration remains within the tissue.

### mSCs maintain their identity during regeneration but show increased plasticity during tumourigenesis

**Fig. 7.**
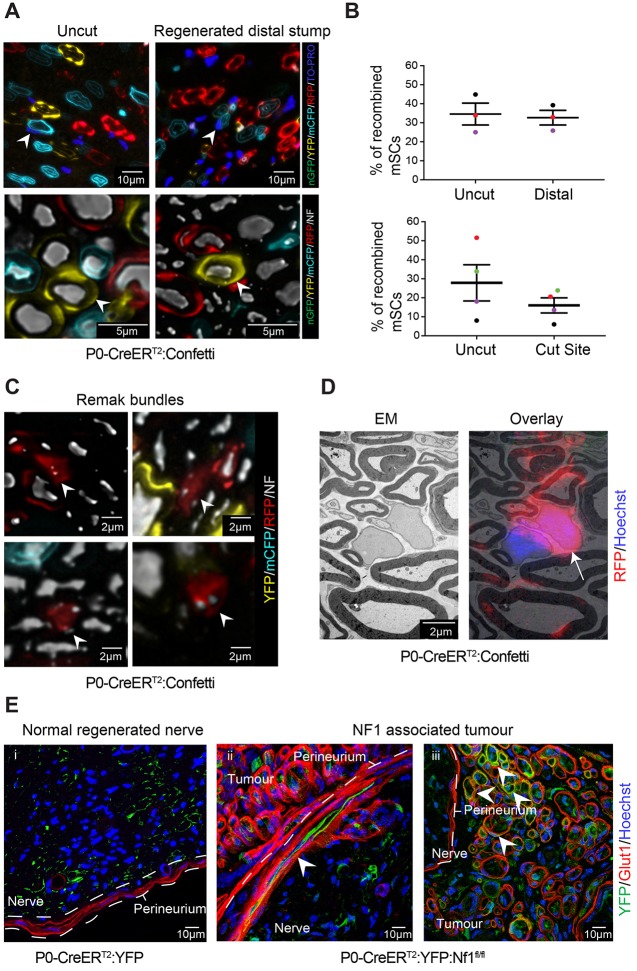
**mSCs retain a SC fate following nerve injury but can switch to become a nmSC.** (A) Representative confocal images of the uncut and regenerated distal stump of *P0-CreER^T2^:Confetti* mice, 6 months following injury. The majority of the labelled mSCs redifferentiated back to mSCs, as seen by their morphology and association with large calibre axons, labelled for neurofilament (NF, white). Arrowheads indicate examples. Nuclei are labelled in the upper row with TO-PRO (dark blue). (B) Graphs show the percentage of recombined mSCs in the distal stump and at the injury site, compared with those in the uninjured contralateral nerve, in *P0-CreER^T2^:Confetti* mice 6 months following injury. Each coloured dot indicates an individual animal. Lines show mean±s.e.m. (C) Representative confocal images of transverse sections of regenerated sciatic nerves of *P0-CreER^T2^:Confetti* mice, showing labelled mSCs that have redifferentiated into nmSCs. Arrowheads indicate labelled Remak bundles associated with small calibre axons (labelled for NF, white). (D) CLEM images of a 200 μm transverse section of sciatic nerve from a *P0-CreER^T2^:Confetti* mouse at Day 90 after nerve transection, showing an RFP-labelled mSC (indicated by arrow) that has redifferentiated to become a nmSC following injury. (E) Representative confocal images showing (i) a regenerated nerve of *P0-CreER^T2^:YFP* mice, in which mSCs retain their lineage and (ii,iii) tumours from *P0-CreER^T2^:YFP:Nf1^fl/fl^* mice, which develop neurofibromas derived from *Nf1^−/−^* mSCs specifically at the injury site. Arrowheads indicate *Nf1^−/−^/YFP^+^* mSC-derived cells, which have taken a different cell fate within the tumour. (ii) A YFP-positive cell, which has integrated within the perineurium. (iii) YFP^+^, perineurial-like cells, which have a distinct morphology and are labelled with Glut1, a marker of perineurial cells. See also Figs S6 and S7.

It has previously been shown that adult SCs retain a level of multipotency (Adameyko et al., 2009; Kaukua et al., 2014; Masaki et al., 2013; Widera et al., 2011). However, these studies were mostly in relatively non-physiological situations, and it has been shown for other cell types that the environmental context can influence cell plasticity (Anderson, 2001). In particular, a recent study showed that the reported multipotency of pericytes *in vitro* was not recapitulated when lineage analysis was performed in the animal (Guimarães-Camboa et al., 2017). To address the plasticity of mSCs, we used lineage analysis to track the fate of mSCs following injury. We then compared the behaviour of normal mSCs following injury with those in a mouse model of NF1 tumourigenesis, in which tumours derive from mSCs (Ribeiro et al., 2013). For the injury studies, we treated either *P0-CreER^T2^:YFP* or *P0-CreER^T2^:Confetti* mice with tamoxifen 14 days prior to injury, and harvested the injured and contralateral uninjured nerves 6 months later. Analysis of these nerves showed that the vast majority of the labelled mSCs redifferentiated to mSCs, as shown by their morphology and association with large calibre axons and P0 staining (Fig. 7A, Fig. S6). Importantly, by pulse labelling mice with a single dose of EdU, 3 days after the injury, we were able to show that mSCs that dedifferentiated and proliferated shortly after injury remyelinated normally, as we could detect YFP^+^/EdU^+^ mSCs within the regenerated nerve (Fig. S6). To determine whether cells derived from mSCs contributed proportionally to the new mSC population in regenerated nerve, we compared the rate of recombination in the contralateral nerves with that in the distal regenerated nerve in mice with lower recombination rates. We found that the proportions remained the same (Fig. 7B), providing strong evidence that an additional stem cell or progenitor population was not contributing to the production of mSCs in the regenerated nerve. In contrast, the percentage of recombined cells was lower in some animals in the region of the cut site (Fig. 7B). This is consistent with our findings that, in some animals, proportionally fewer mSCs migrated into the bridge region compared with those derived from nmSCs (Fig. 4E).

To determine whether cells derived from mSCs retained any plasticity, we initially addressed whether these cells could become nmSCs. We analysed the new tissue of the bridge region, where the loss of the Bands of Büngner may permit greater contact with smaller axons, and found several examples of labelled SCs that had redifferentiated to become nmSCs, as shown by their association with small calibre axons in the characteristic structure of a Remak bundle (Fig. 7C). We also found these cells within the distal stump region. To confirm these findings, we performed correlative light and EM (CLEM) and found multiple examples of labelled Remak bundles in the regenerated nerves (Fig. 7D, Fig. S7). These findings show definitively that mSCs retain the plasticity to become nmSCs following an injury. Moreover, the finding that there was a decrease in the proportion of labelled cells in the bridge region of the cut (Fig. 7B) suggests that SCs derived from nmSCs make a contribution to the regenerated mSC population in this new tissue. Together, these findings indicate that dedifferentiated SCs from both the nmSC and mSC population are able to switch between their respective roles.

In contrast to this limited plasticity, we did not find a single example of a mSC becoming another cell type within the regenerated nerve. These findings suggest, as has been shown for other cell types, that the reported plasticity of cells is the result of the non-physiological environment in which the experiments were performed, and that the microenvironment is important for maintaining cell identity (Guimarães-Camboa et al., 2017; Snippert and Clevers, 2011). Consistent with this view, we found a different scenario in the tumour mouse model (*P0-CreER^T2^:YFP:Nf1^fl/fl^*), in which, following an injury, tumours derived from mSCs only develop at the injury site (Ribeiro et al., 2013). Analysis of these nerves showed that, although cells derived from *Nf1^−/−^* mSCs mostly redifferentiated into mSCs or became tumour cells, a proportion appeared to become other cell types (Fig. 7E). These included a large number of cells that expressed Glut1 and resembled perineurial-like cells within the tumour mass (Fig. 7E). Moreover, we found several examples of YFP^+^ cells that had integrated into the perineurium (Fig. 7E). These cells were never seen in control animals or in the distal stump of *P0-CreER^T2^:YFP:Nf1^fl/fl^* animals. These findings demonstrate that SCs retain their identity within the context of a regenerating nerve and exhibit limited plasticity, whereas a tumourigenic genetic change and the microenvironment can synergise to increase the plasticity of these cells.

## DISCUSSION

It is becoming increasingly clear that different tissues have distinct mechanisms to maintain themselves in the adult and to repair following an injury (Ge and Fuchs, 2018; Varga and Greten, 2017; Wells and Watt, 2018). In proliferative tissues, the classical model of a defined stem cell population dividing infrequently to produce a transiently amplifying population, which then differentiates to produce the various cells of a tissue, has been supplanted by more diverse models. These include stem cells that divide frequently, reserve stem cell compartments, the ability of committed cells to dedifferentiate to form stem cells and separate stem cell compartments that are activated upon injury. Moreover, the mechanism to produce new cells often varies dramatically between the homeostatic, injured and repairing states.

Here, we have systematically characterised the cells that make up the endoneurium of peripheral nerves, although we cannot rule out a failure to detect rare cells that are not labelled by any of our methodologies. We find that it is a highly quiescent tissue, with most cell types proliferating rarely, while the main cell type of the nerve, the mSC, does not divide at all in the adult. The stability of this tissue is presumably possible because peripheral nerves, once formed and matured, tend to retain their structure and their connections. Moreover, peripheral nerves are protected by the BNB, which perhaps contributes to the low turnover rate of cells within this tissue. However, once injured, all cell types within a peripheral nerve are able to re-enter the cell cycle and do so at very high efficiency, apparently obviating the requirement for a stem cell population to produce new cells for the regenerative process. This switch from a highly quiescent tissue to a highly proliferative tissue – with all cell types contributing to the regenerative response – represents a further model of how tissues maintain and repair themselves.

In this study, we have also characterised a new cell type. These cells are NG2^+^/PDGFRβ^+^, the classical markers for pericytes. However, although these cells are found loosely associated with blood vessels, they are not found within the basal lamina and are negative for αSMA (unlike all the pericytes detected in peripheral nerve). They are also positive for p75^+^, a marker for dedifferentiated SCs and nmSCs, but negative for S100, and are not marked in any of our SC-specific lineage-tracing mice or in the *PLP-eGFP* mice. They also have a unique morphology, with long protrusions spreading throughout the endoneurium, which make apparent contact with the other cell types within the nerve. Because of their extensive endoplasmic reticulum, they have previously been referred to as fibroblasts; however, because of their unique set of markers and distinct morphology, we propose to call them tactocytes (touching cells). They make up a substantial proportion (12.5%) of the cells within a peripheral nerve and future studies are needed to uncover their roles in nerve function.

We have characterised the behaviour of the mSC in most detail, which has been possible because of a highly specific driver for this cell type in adulthood that permits credible lineage analysis. These studies show that the mSC is a truly remarkable cell. We were unable to detect a single proliferating mSC throughout the nerves of multiple animals, which indicates that, once formed, these highly specialised cells do not turn over. However, these cells are not postmitotic. Using lineage analysis, we were able to show that, following an injury, close to 100% of mSCs proliferated within a few days of the injury. Moreover, they showed a dramatic change in their behaviour to become the migratory cells that transport regrowing axons across the injury site. The ability of all mSCs to proliferate following an injury would seemingly indicate the lack of need for a further stem cell population to produce new SCs during the regeneration of peripheral nerves. This view is further substantiated by lineage analysis of animals with lower levels of recombination, in which we found a similar proportion of recombined mSCs in an individual animal's uncut and contralateral regenerated sciatic nerve, showing that the original population is not diluted by an influx of stem cells from another source. It could thus be argued that there is not a stem cell/progenitor population to produce new mSCs in the adult, or that all mSCs have the capacity to act as stem/progenitor cells. The latter argument is augmented by studies showing that dedifferentiated SCs have unlimited proliferative capacity (Mathon et al., 2001) and retain the ability to redifferentiate back to a more differentiated cell state.

It is particularly striking that very different mechanisms are used to maintain the myelinating cells of the PNS and CNS. New OLs are produced throughout life. Although much of this is thought to be to myelinate new axons, and is associated with learning and memory, there also appears to be a higher turnover of mature cells, as axons in the optic nerve are fully myelinated yet appear to turn over throughout adulthood (Young et al., 2013). Why mSCs are more stable than OLs in the optic nerve is not clear, as the environments appear similar in that they are both stable structures and are protected by the BNB and blood-brain barrier, respectively. The mechanisms to produce new myelinating cells are also different. There only appears to be a requirement to produce new mSCs following injury, as normally these cells do not turn over in the adult. However, following injury, new cells are produced by the dedifferentiation and proliferation of the mSCs themselves. In contrast, the CNS is populated throughout by precursor cells (OPCs), which proliferate throughout adulthood to maintain themselves and differentiate throughout life to produce new OLs (Birey et al., 2017). Why such differences exist can only be speculated upon, but they are likely to reflect a trade-off between the increased plasticity required by the CNS versus the stability required by the PNS. The presence of a continually proliferating progenitor population whilst allowing a rapid source for new myelination also provides a pool susceptible to tumour development. Consistent with this, malignant tumours are more frequent in the CNS than in the PNS, which perhaps reflects the presence of a more susceptible proliferating progenitor population.

The lineage analysis used in this study indicates that, during the regenerative process, SCs retain their identity. Studies have suggested that SCs retain multipotency, but, similar to findings from *in vivo* studies in other tissues, it appears that the normal tissue environment restricts plasticity (Anderson, 2001; Guimarães-Camboa et al., 2017). We found that dedifferentiated mSCs, while retaining a SC identity, were able to become nmSCs, showing that these cells retained the ability to respond to the axonal environment and choose between a myelinating or non-myelinating fate. However, we found that this lineage restriction breaks down in the context of tumourigenesis. This required both a tumourigenic mutation (loss of *Nf1*) and a conducive microenvironment. However, these findings show that increased plasticity can be induced in the SC lineage, which potentially has implications for the pathology of these tumours.

Peripheral nerves regenerate even following a full transection and functionality can be restored, in contrast to the poor regenerative capability of the CNS. However, our studies, and others, show that a regenerated nerve differs markedly from an uninjured nerve (Napoli et al., 2012; Salonen et al., 1988; Zochodne, 2008). This includes a large increase in the cellularity of regenerated nerve. Notably, the relative proportion of all cell types remained the same, indicating that an unknown homeostatic mechanism exists to ensure the structure of the nerve. However, the major difference appears to be increased ECM levels in regenerated nerve. ECM deposition is a key aspect of an injury response (Eming et al., 2017), and it is likely that the failure to clear the injury-induced ECM contributes to the inability of repaired tissue to return to the uninjured state. Targeting the clearance of accumulated ECM could thus provide a strategy for improving tissue repair.

In summary, we report that peripheral nerve provides a further example of the diverse mechanisms by which tissues maintain themselves and repair following injury. In the adult, peripheral nerve is highly quiescent yet retains function throughout adulthood. However, despite this stability, it has remarkable regenerative properties without the need for a specialised stem cell compartment – instead all the cell types of the nerve are able to proliferate to contribute to the regeneration of this tissue.

## MATERIALS AND METHODS

### Transgenic mice

All animal work was performed in accordance with UK Home Office legislation. Mice were housed in a temperature- and humidity-controlled vivarium on a 12-h light-dark cycle with free access to food and water. Female and male (4-week- to 1-year-old) mice of the following genotypes and strains were used: for lineage tracing of mSCs, *P0-CreER^T2^* C57Bl/6 mice (Leone et al., 2003; Ribeiro et al., 2013) were crossed with *R26R-YFP* (Srinivas et al., 2001), *R26R-tdTomato* (Madisen et al., 2010) or *R26R-Confetti* reporter mice (Livet et al., 2007; Snippert et al., 2010) to generate *P0-CreER^T2^:YFP*, *P0-CreER^T2^:tdTomato* and *P0-CreER^T2^:Confetti* mice. To visualise all SCs, *P0-Cre* mice (Feltri et al., 1999) were crossed with *R26R-tdTomato* mice to generate *P0-Cre:tdTomato* and *Plp-eGFP* transgenic mice (Mallon et al., 2002) were used. *NG2-dsRed* mice were used to confirm the identity of NG2^+^ cells (Zhu et al., 2008). To distinguish between NG2^+^/PDGFRβ^+^/p75^+^/αSMA^−^ and dedifferentiated SCs within the nerve bridge, *Plp-eGFP* mice were crossed with *NG2-dsRed* mice to generate *PLP-eGFP:NG2-dsRed* mice. For genotyping primer sequences used in this study, see Table S3.

For studying SC plasticity in a nerve tumour environment, *P0-CreER^T2^:YFP:Nf1^fl/fl^* mice were used (Ribeiro et al., 2013). In all studies, both male and female mice were used. Animals were genotyped and identified using earhole punches upon weaning. For further details of the transgenic mice used in this study, see Table S2. For lineage-tracing experiments, Cre-mediated recombination was induced in 4- to 5-week-old mice by intraperitoneal injection (IP) or oral administration of 2 mg tamoxifen (Sigma-Aldrich, H7904) daily for 5 consecutive days. Tamoxifen was dissolved in sunflower oil (20 mg/ml) and filtered through a 0.2 μm filter.

### Sciatic nerve injury

Fourteen days after tamoxifen administration, mice were anaesthetised with isofluorane under aseptic conditions and the right sciatic nerve was exposed at the sciatic notch. The nerve was fully transected or half transected, as indicated, and the wound closed with clips. Nerves were then collected at the indicated days for analysis by immunostaining or EM.

### EdU administration

To cumulatively label newly generated cells, adult c57bl/6 or *PLP-eGFP* mice received 0.2 mg/ml EdU (Thermo Fisher Scientific, A10044) in their drinking water for up to 70 days as indicated (Young et al., 2013). The water was changed every 48 h. For pulse-labelling experiments, a single EdU injection (2 mg of EdU in PBS) was given by IP 3 h prior to tissue collection. Cell proliferation was determined by measuring EdU incorporation detected using the Click-iT™ EdU kit (Thermo Fisher Scientific, C10339) according to the manufacturer's instructions.

### IF staining

Sciatic nerves were dissected and fixed for 4 h in 4% paraformaldehyde (TAAB) at room temperature and embedded for cryosectioning. For immunostaining, pre- or postfixed longitudinal sections of the sciatic nerves were immunostained as detailed in the Supplementary Materials and Methods and analysed using confocal microscopy.

### Primary antibodies

The following primary antibodies were used for IF at the indicated dilutions: anti-P0 (1/500, Abcam, ab39375), anti-p75 (1/500, Millipore, ab1554), anti-S100 (1/1000, Dako, Z0311), anti-Iba1 (1/500, Wako, 019-19741), anti-CD31 (1/100, BD Biosciences, 553370), anti-neurofilament 200 kD (1/1000, Abcam, ab4680), anti-laminin (1/500, Abcam, 11575), anti-collagen III (1/1000, Abcam, ab7778), anti-fibronectin (1/500, Sigma-Aldrich, clone FN-3E2), anti-NG2 (1/500, Millipore, ab5320), anti-PDGFRβ (1/500, Abcam, ab32570), anti-αSMA (1/1000, Sigma-Aldrich, C6198), anti-GFP (1/1000, Abcam, ab13970), anti-Glut1 (1/500, Abcam, ab652), anti-F4/80 (1/100, Bio-Rad, MCA497G) and anti-NG2 (1/100, Thermo Fisher Scientific, MA5-24247). For further details of primary antibodies used in this study, see Table S1.

### Confocal microscopy

Confocal images were acquired using an inverted SPE or SP8 confocal microscope (Leica). A multiphoton microscope (Zeiss) was used to image confetti samples. Within each experiment, the same acquisition settings were used, the same volume imaged and the same number of *z*-stacks acquired. Fiji software (https://imagej.net/Fiji/Downloads) was used to make a projection of the *z*-stacks. For 3D projections, Imaris software was used (http://www.bitplane.com/imaris).

### Transmission EM

Sciatic nerves were dissected and fixed overnight at 4°C in 2% glutaraldehyde in 0.2 M phosphate buffer. Nerves were then postfixed in 2% osmium tetroxide for 1.5 h at 4°C, and incubated in 2% uranyl acetate for 45 min at 4°C. Nerves were dehydrated in an ethanol series before being incubated with propylene oxide and embedded in epoxy resin. Ultrathin sections (70 nm) were cut with a diamond knife (Diatome), collected onto formvar-coated slot grids and visualised using a transmission electron microscope (T12 Tecnai Spirit, FEI) using a Morada camera and iTEM software (Olympus SIS). Semithin sections were cut using a diamond Histo knife (Diatome) at 0.2 μm, dried and stained with 1% Toluidine Blue in 2% Borax at 75°C for 2 min. Dried sections were mounted with DPX (Sigma-Aldrich) and representative images of the entire nerves were acquired using a wide-field microscope (Zeiss Axio Scope.A1).

### CLEM

Regenerated sciatic nerves from *P0-CreER^T2^:Confetti* mice were harvested 3 months following injury and fixed in antigenfix (Diapath) overnight. The following day, nerves were embedded in 2.8% low melting point agarose in PBS. Sections (200 µm) of the embedded nerve were cut in cold PBS using a vibrating microtome. For CLEM analysis, sections were first analysed using a SP8 confocal microscope (Leica) and then processed for EM. For further details of the CLEM protocol used in this study, see the Supplementary Materials and Methods.

### Quantification and statistical analysis

For details of the image quantification analysis used in this study, see the Supplementary Materials and Methods.

### Statistical analysis

Statistical and graphical data analyses were performed using Prism 7 (GraphPad). For all measurements, three or more biological replicates were used. Information regarding the number of biological replicates (‘*n*’) used in each experiment is reported in the relevant figure legends. The data are presented as mean± s.e.m. Unpaired two-tailed Student's *t*-test was used for statistical analysis, except where ANOVA is indicated. *P<0.05, **P<0.01, ***P<0.001.

## Supplementary Material

Supplementary information
