# Supplementary material for: The regulation of the homeostasis and regeneration of peripheral nerve is distinct from the CNS and independent of a stem cell population
Source: Development. 2018 Dec 14;145(24):dev170316. doi: 10.1242/dev.170316 (PMC6307893; doi:10.1242/dev.170316)
Supplement: Supplementary information [file develop-145-170316-s1.pdf]

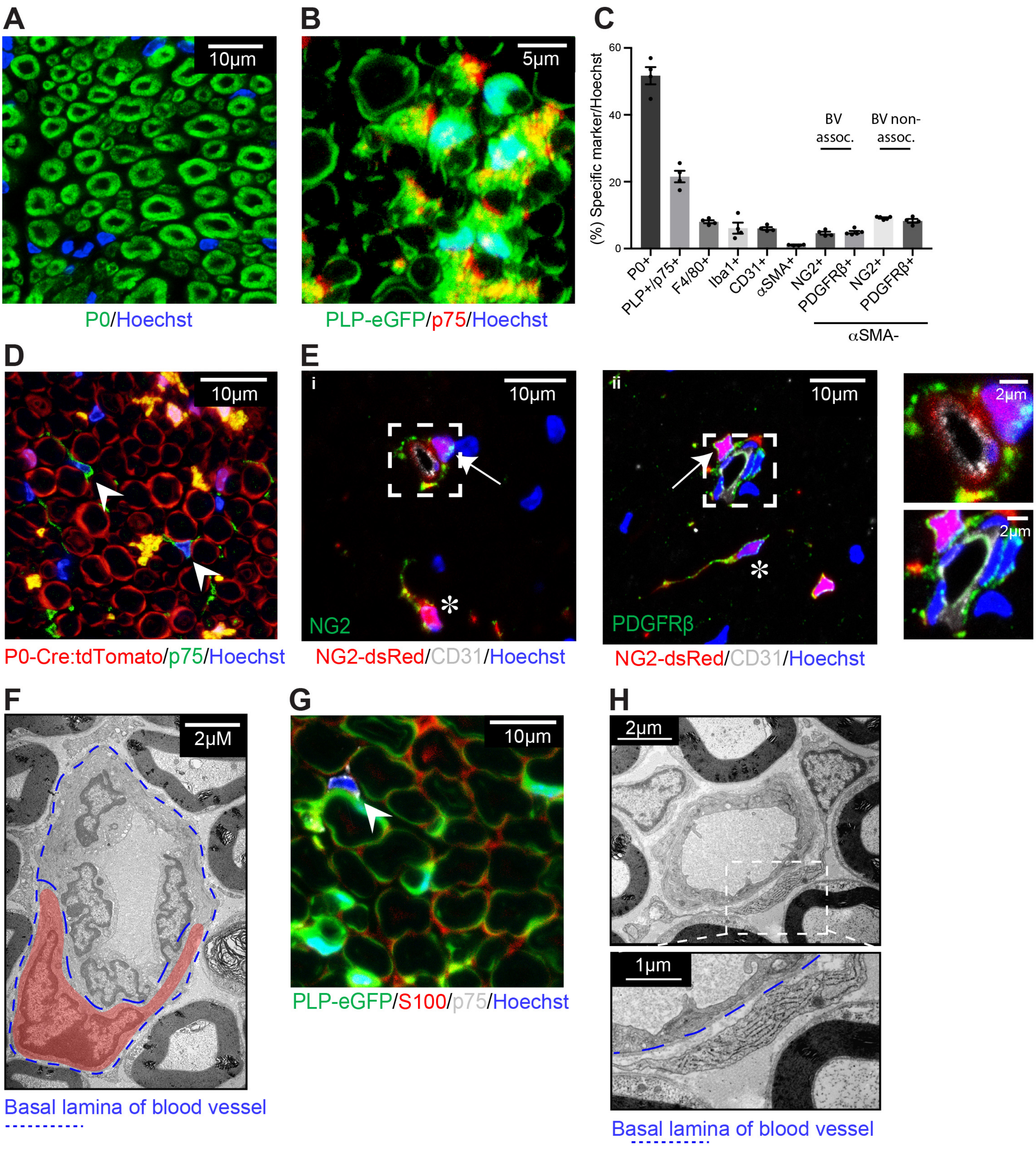

Figure S1, related to Figure 1. Identification of the cell composition of peripheral nerve. Representative confocal images of **A)** mSCs (P0+) and **B)** nmSCs (PLP+/ p75+). **C)** Graph shows the quantification of specific markers expressed by cells in sciatic nerve (n=4-5 mice, mean±SEM). Pericytes are defined by the co-expression of NG2, PDGFRß and αSMA. A separate population is NG2/ PDGFRß+ but αSMA-. These cells were further characterised as associated or non-associated with CD31+ blood vessels (BV). **D)** Representative confocal image shows a transverse section of a sciatic nerve isolated from a P0-Cre:tdTomato mouse immunostained for p75 (green). Arrowheads indicate tdTomato-/ p75+ cells. **E)** Sections from NG2-dsRed mice were co-stained for (i) NG2 (green) and (ii) PDGFRß (green). Arrows indicate CD31+ - associated cells, asterisks indicate a CD31+ non-associated population. White rectangles indicate the regions that are shown at higher magnifiation on the right. **F)** Representative EM image showing a classical pericyte (red), which is in tight contact with a blood vessel and is within the basal lamina. **G)** Transverse section of a sciatic nerve isolated from a PLP-eGFP mouse immunostained for p75 (grey) and S100 (red). Arrowhead indicates an eGFP-/S100-/p75+ cell. **H)** Representative EM images show an elongated cell, rich in endoplasmic reticulum, closely associated with a blood vessel. The higher magnification image shows that it is not surrounded by the basal lamina.

Development • Supplementary information

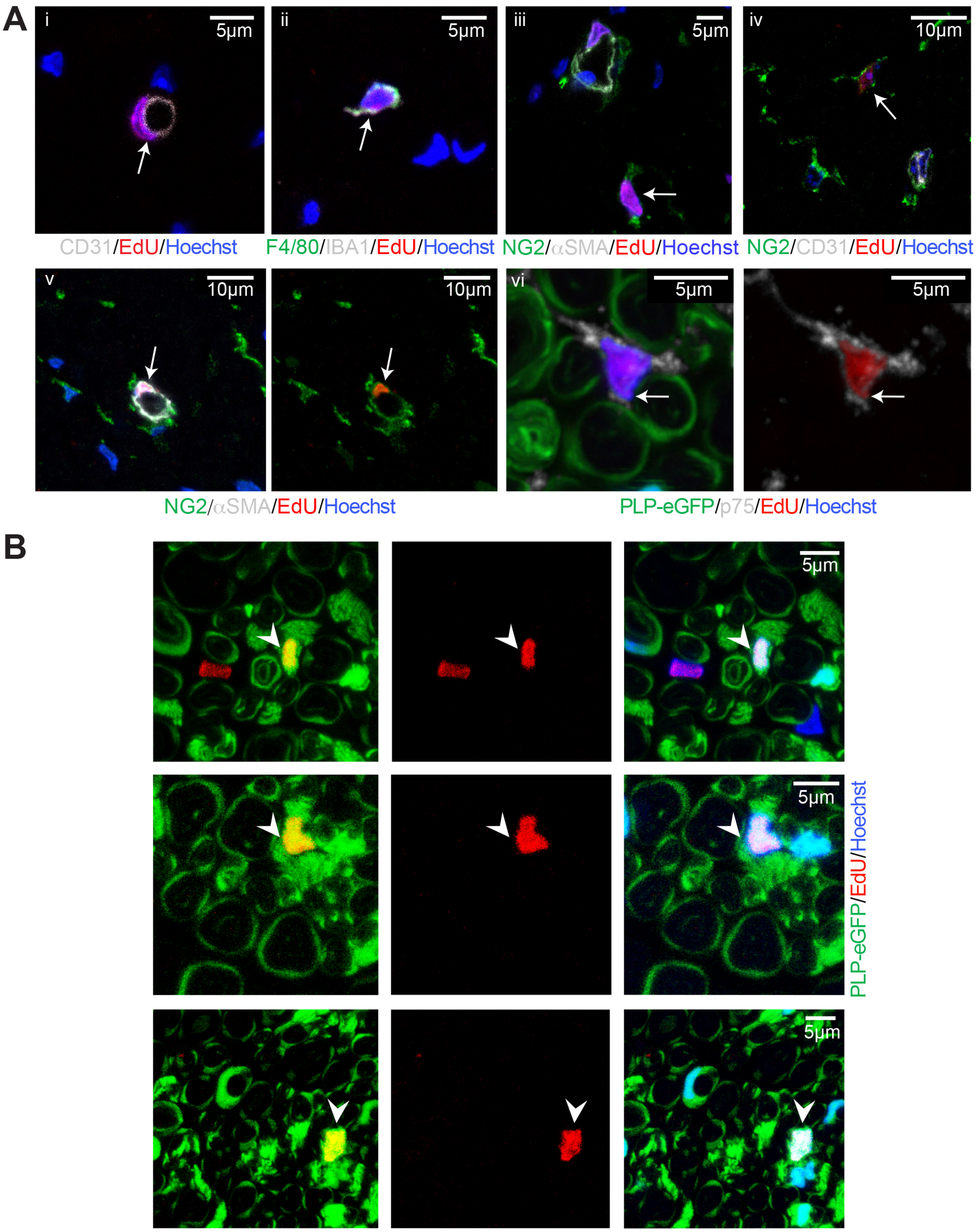

Figure S2, related to Figure 2. Peripheral nerve is a highly quiescent tissue.
A) Representative confocal images of (i-v) transverse sections of sciatic nerve from WT mice and (vi) PLP-eGFP mice treated continuously with EdU for 30 days, processed to detect EdU (red) and immunolabelled for (i) CD31, to detect endothelial cells (white). (ii) F4/80 (green)/ Iba1 (white), to detect macrophages. (iii) NG2 (green) and αSMA (white) to detect pericytes. (iv) NG2 (green), and CD31 (white) to detect NG2+ cells non-associated with CD31+ blood vessels. (v) NG2 (green) and αSMA (white). (vi) p75 (white) to detect PLP-/ p75+/ EdU+ cells. Nuclei are stained with Hoechst (blue). Arrows indicate proliferating cells. **B)** Confocal images of 20µm cryosections of sciatic nerve isolated from PLP-eGFP mice treated continuously with EdU (red) for 30 days. Nuclei are stained with Hoechst (blue). Arrowheads indicate three separate examples of proliferating nmSCs (PLP+/ EdU+).

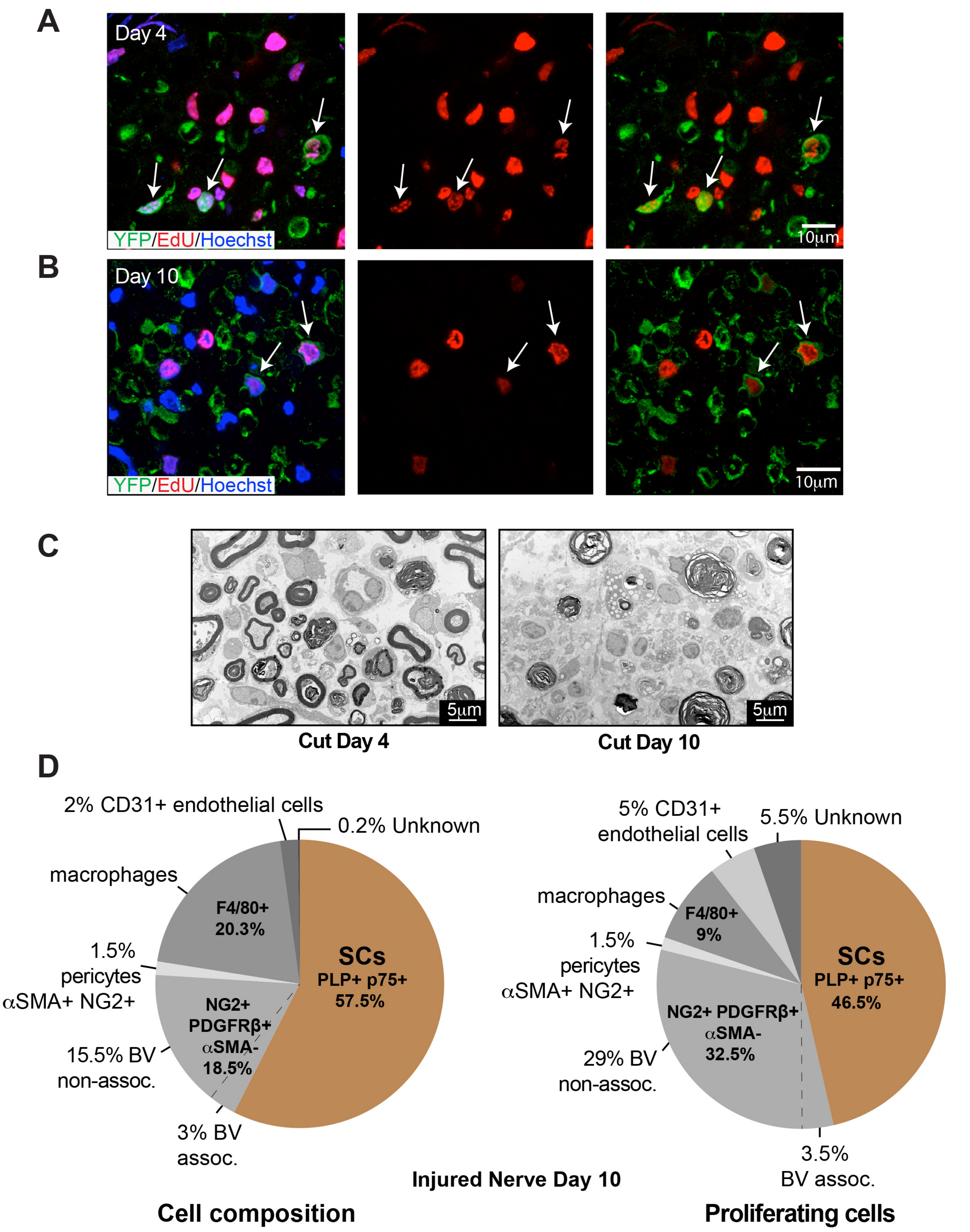

Figure S3, related to Figure 3. All mSCs proliferate following injury.
A-B) Representative confocal images of transverse sections of transected sciatic nerve of P0-CreER^{T2}:YFP mice in which mSCs were labelled with YFP following Tmx administration. Arrows indicate Edu+/ YFP+ cells **A)** Day 10 following injury, EdU was administered continuously in the drinking water. **B)** Day 4 following injury, EdU was injected 3 hours prior to harvesting. **C)** Representative EM images of the distal stump of injured sciatic nerves at Day 4 and Day 10 following transection. **D)** Pie charts show the cell composition and the relative proportions of the cell types proliferating in the distal stump of an injured sciatic nerve at Day 10 following injury (n=8 mice).

Development • Supplementary information

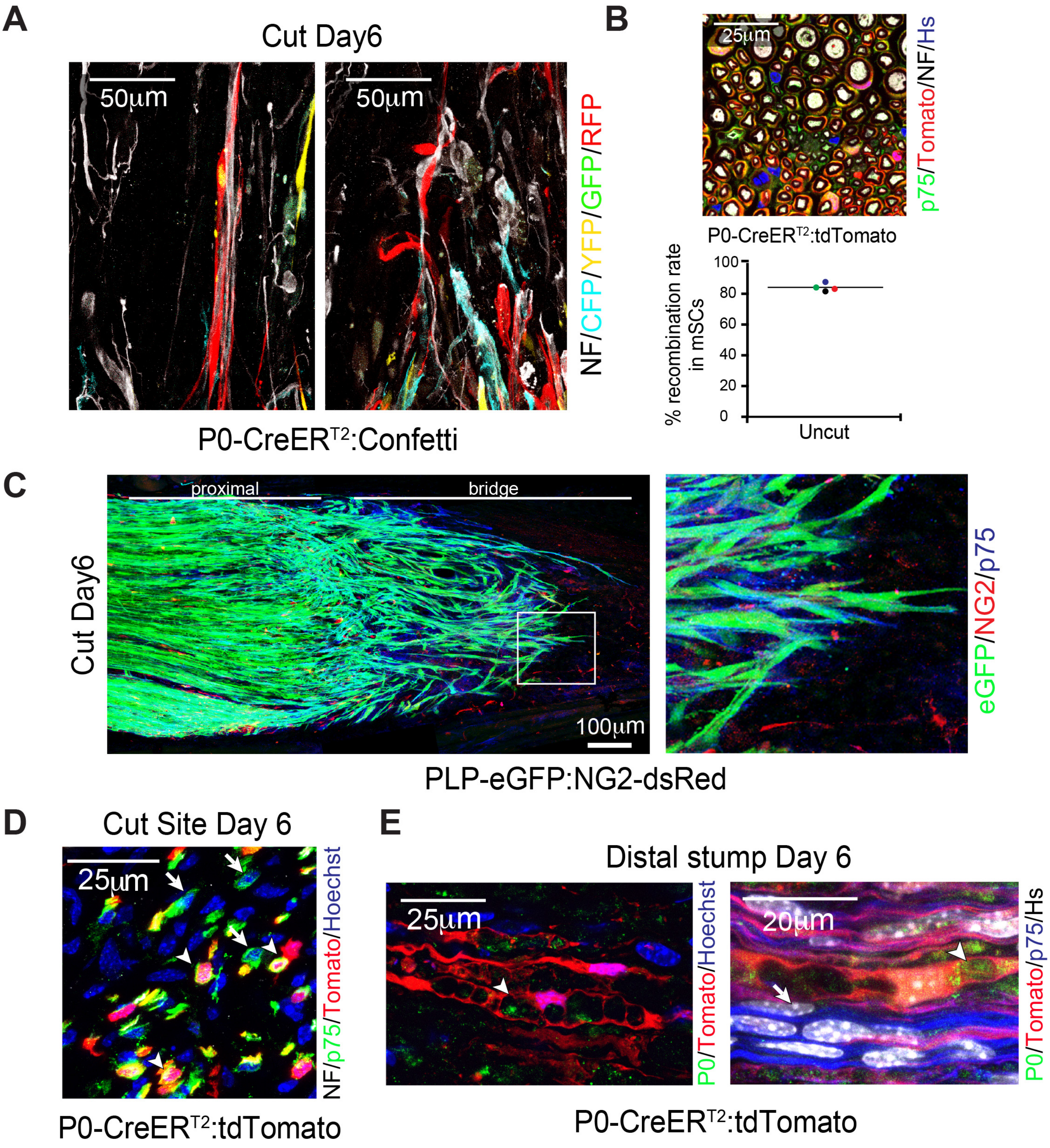

Figure S4, related to Figure 4 : mSCs become migratory following injury to guide regrowing axons.
A) Representative images of a longitudinal cryosection of an injured sciatic nerve of P0-CreER^{T2}:Confetti mice, Day 6 following injury, shows migrating SCs derived from mSCs associated with regrowing axons in the nerve bridge. An antibody to neurofilament (NF) was used to label the axons. **B)** Representative image of a transverse cryosection of uninjured sciatic nerves of P0-CreER^{T2}:tdTomato mice following tamoxifen administration. Antibodies to S100 and neurofilament (NF) were used to label SCs and axons, respectively. Graph shows the percentage of recombined mSCs and shows that more than 80% of mSCs express Tomato following tamoxifen administration. Each coloured dot represents an individual animal. Line shows mean±SEM. **C)** Representative image of a longitudinal cryosection of the bridge region (Cut site) of an injured sciatic nerve from PLP-eGFP:NG2-dsRed mice, Day 6 following injury. An Antibody to p75 was used to label dedifferentiated SCs. **D)** Representative image of a transverse cryosection of the bridge region (Cut site) of an injured sciatic nerve from P0-CreER^{T2}:tdTomato mice, Day 6 following injury. Antibodies to p75 and neurofilament (NF) were used to label dedifferentiated SCs and axons, respectively. Arrowheads indicate Tomato+/p75+ cells and arrows Tomato-/p75+ cells and show that SCs derived from mSCs are associated with axons in the bridge region. **E)** Representative images of a longitudinal section of the distal stump of P0-CreER^{T2}:tdTomato mice, showing that SCs derived from mSCs engulf and degrade myelin debris. Antibodies to P0 and p75 weres used to label dedifferentiated SCs and the degraded myelin, respectively. Arrowheads indicate myelin debris that has been engulfed by myelinating Schwann cell derived cells and the arrow indicates a Tomato-/P75+ cell devoid of myelin debris.

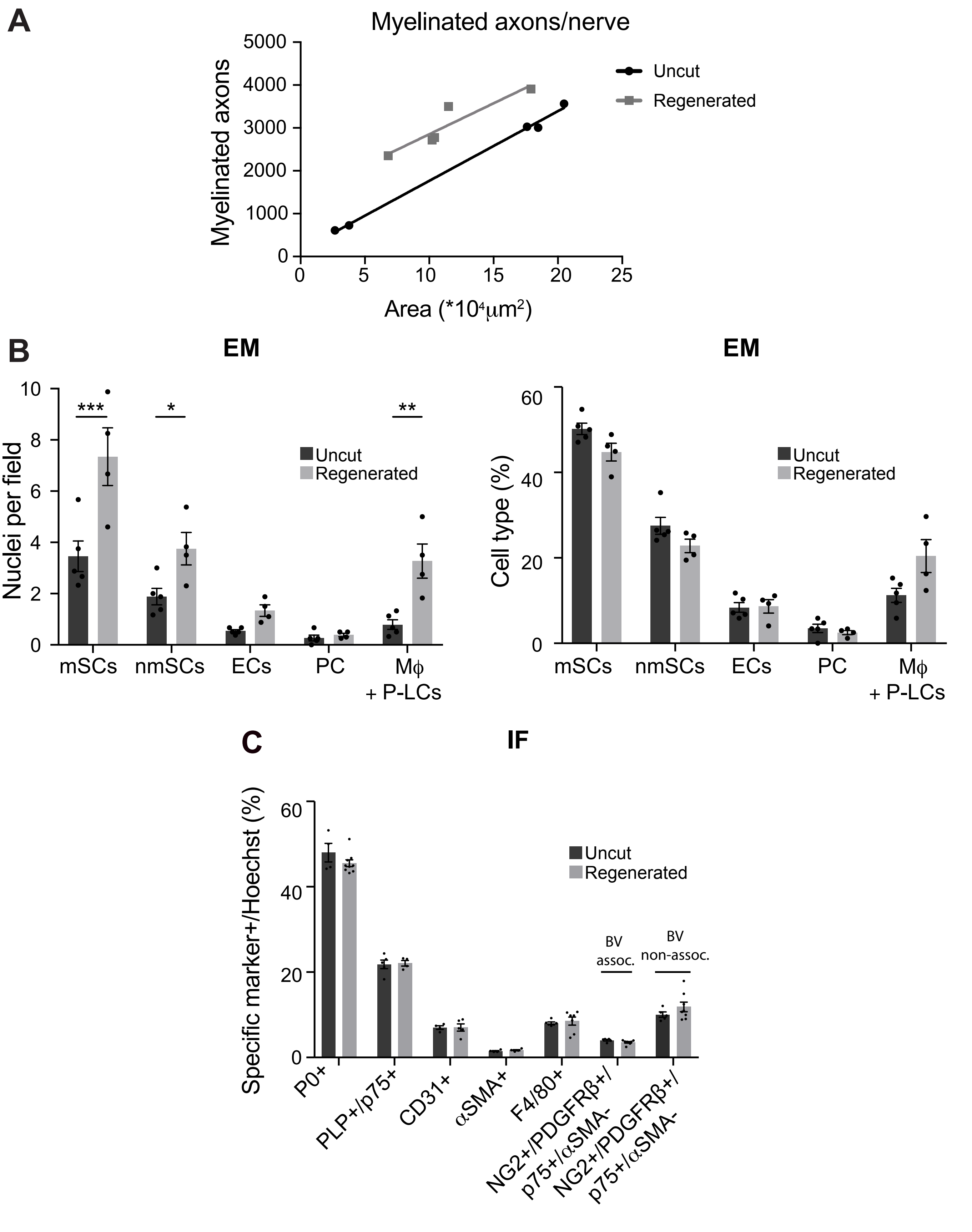

Figure S5, related to Figure 5. Regenerated nerve is distinct from uninjured nerve.
A) Graph shows the quantification of the total number of axons in uncut and regenerated nerve. Each point represents an individual nerve. **B)** Quantification of EM images showing the number of specific cell types per field (LHS) and the proportion of each cell type (RHS) in uncut and regenerated nerves, 6 months following injury. Cells were identified by morphology. EC (endothelial cells), PC (pericytes). We were not confident distinguishing macrophages (Mϕ) from NG2+/ PDGFRβ/ P75+ cells, labelled as P-LCs (pericyte-like cells) in the EM quantifications, and so these cells were grouped together (n=4-5 mice, mean±SEM, two-way ANNOVA was used). **C)** Quantification of immunofluorescent images showing the proportion of each cell type, identified by the indicated markers, in the uncut and regenerated nerves, 6 months following injury (n=4-9 mice, mean±SEM).

Development • Supplementary information

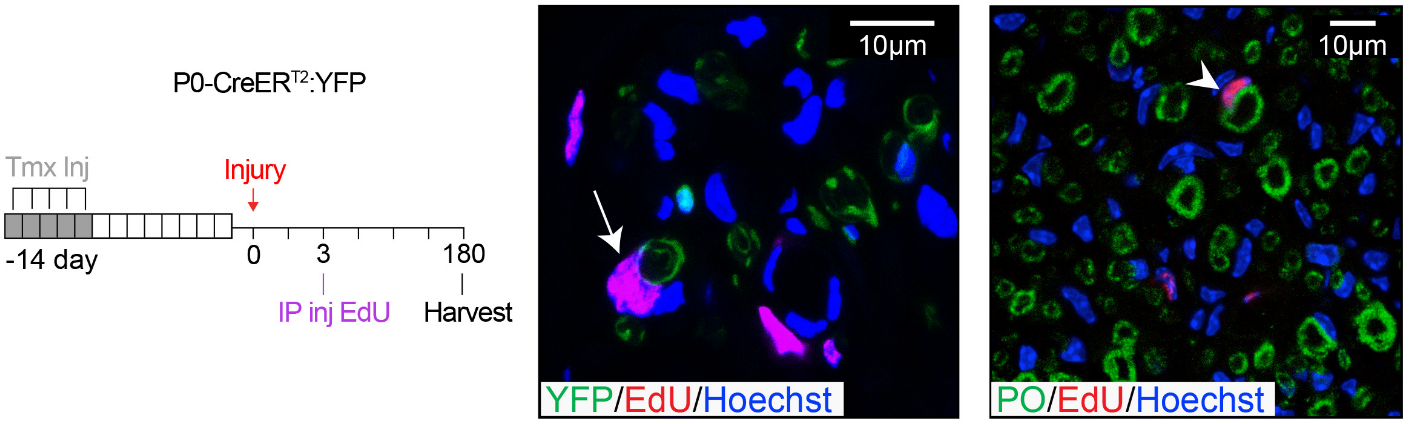

Figure S6, related to Figure 7. mSCs retain a SC fate following nerve injury but can switch to become a nmSC.

Schematic representation of the protocol used to determine whether proliferating cells derived from mSCs remyelinate axons. P0-CreER^{T2}:YFP mice were injected intraperitoneally with Tmx for 5 consecutive days. 14 days following the first injection, the right sciatic nerve was cut and EdU was injected at Day 3 following injury. Mice were harvested 6 months later. Representative confocal images show transverse sections from the regenerated nerve of EdU injected P0-CreER^{T2}:YFP mice, 6 months following injury. The arrow indicates a YFP+ mSC that proliferated at Day 3 following injury and redifferentiated into a mSC. The arrowhead indicates a cell that proliferated at Day 3 following injury and became a mSC, as indicated by P0 staining.

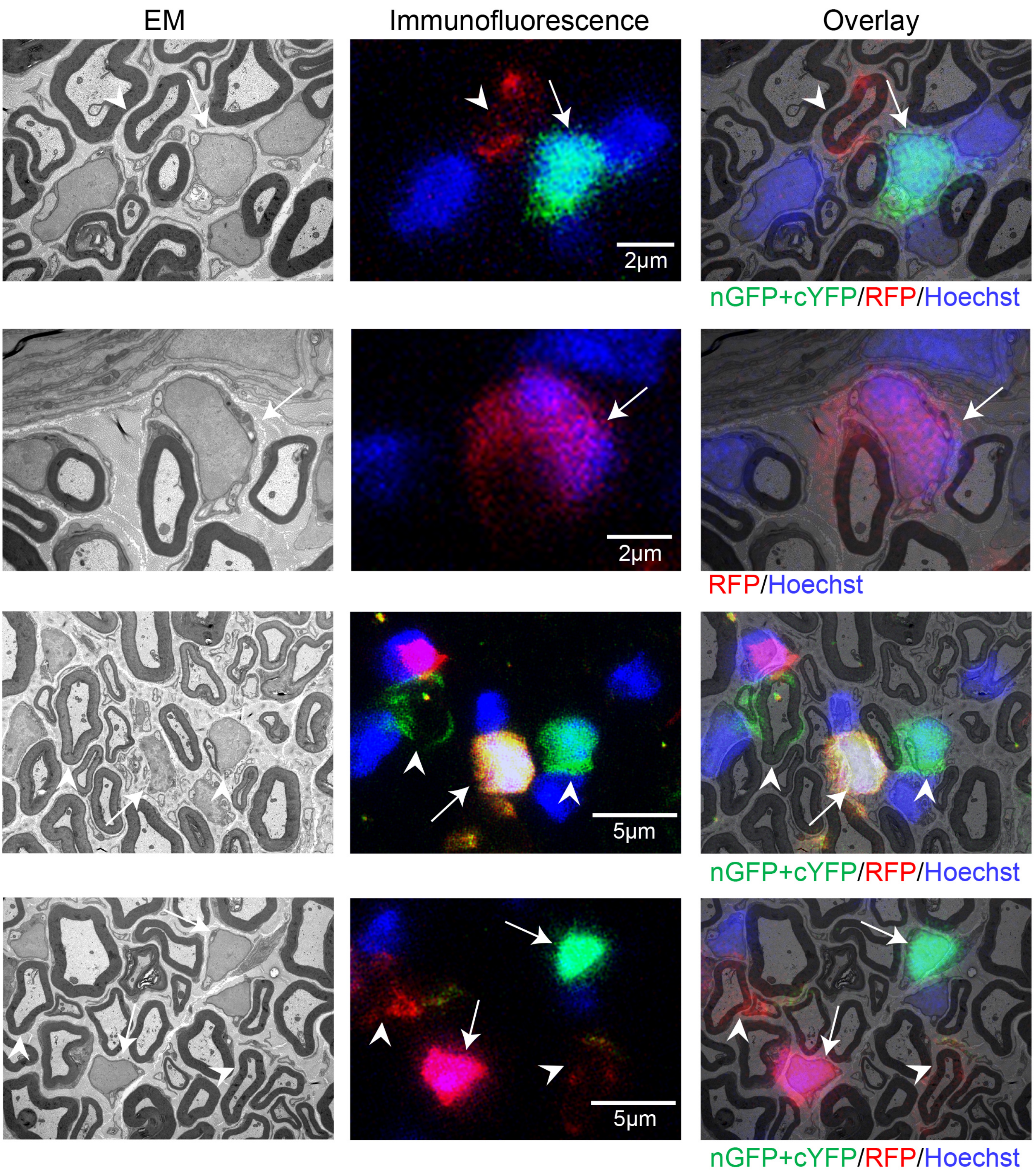

Figure S7, related to Figure 7. mSCs retain a SC fate following nerve injury but can switch to become a nmSC.
A series of Correlative Light and Electron Microscopy (CLEM) images of 200µm transverse sections of sciatic nerve isolated from P0-CreER^{T2}:Confetti mice, Day 90 after nerve transection, showing cytoplasmic RFP (red), nuclear GFP and cytoplasmic YFP (green) labelled mSCs that have redifferentiated to become nmSCs following injury. Panels show the EM image (left), the corresponding confocal image (middle) and the overlayed images (right). Arrowheads indicate labelled mSCs and arrows indicate mSC-derived cells that have differentiated into nmSCs.

Supplementary Information

Supplementary Materials and Methods

Immunostaining

After fixation in PFA, nerves were cryoprotected in 30% sucrose (w/v) in PBS overnight at 4ºC. The nerves were then transferred to a 1:1 mixture of 30% sucrose with O.C.T compound (TissueTek, Sakura) for 2 hours at RT and finally embedded in O.C.T before being snap-frozen in liquid nitrogen. 10-25µm longitudinal or transverse cryosections (Leica) were permeabilised in 0.3% Triton-X100 in PBS for 30 min, blocked in 10% goat serum/PBS for ≥1 hour and incubated in primary antibodies diluted in blocking buffer overnight (O/N) at 4°C. Sections were washed 3 times with PBS and the appropriate fluorescent secondary antibodies were used with Hoechst to counterstain the nuclei for 1h at RT. Samples were mounted in Fluoromount G (Southern Biotechnology) before imaging. Sciatic nerves from P0-CreER^{TR}:Confetti mice were fixed in Antigenfix (DiaPath) to preserve the endogenous fluorescence and RedDot (Biotium) or To-Pro-3 (Thermo Fisher Cat# T3605) was used as a nuclear counterstain. For better preservation of axonal proteins, PBS containing 1mM $CaCl_2$, 0.5mM $MgCl_2$ was used when needed. For P0 staining, harvested sciatic nerves were instead immediately snap frozen in liquid nitrogen. After cutting 10-25µm transverse cryosections, the sections were postfixed for 10min using 4% PFA at RT and washed thoroughly with PBS before blocking with 10% goat serum/PBS. The rest of the immunostaining protocol was as the staining protocol for the prefixed nerve.

Correlative light and electron microscopy (CLEM)

200µm vibrotome sections were screened using a widefield fluorescence microscope to identify sections containing a large number of fluorescently-labelled cells. These sections were imaged with a 40x lens using a SP8 confocal microscope (Leica). YFP, GFP and RFP was acquired (CFP excitation is not possible on this microscope. After image acquisition, samples were fixed in 2% (wt/vol) PFA, 1.5% (wt/vol) glutaraldehyde (both EM grade from TAAB) in 0.1M sodium cacodylate buffer for 30 mins at RT. Samples were then secondarily fixed in 1% (wt/vol) osmium tetroxide, 1.5% (wt/vol) potassium ferricyanide for 1h at 4 °C. After washes in 0.1M sodium cacodylate, samples were incubated in 1% (wt/vol) tannic acid in 0.5M sodium cacodylate at room temperature for 45 min. Further washes in 0.5M sodium cacodylate were followed by a final wash in distilled water, before the samples underwent dehydration by sequential short incubations in 70% (vol/vol) and 90% (vol/vol) ethanol and then two longer incubations in 100% ethanol. Samples were transferred to a 1:1 mix of propylene oxide and Epon resin (TAAB) for 90 min, then 100% Epon for two more incubations, one of several hours and one O/N. Finally samples were polymerised by baking at 60°C O/N. It is important that the side of the sample that was imaged by confocal microscopy faces the top of the

Development • Supplementary information

resulting resin block to ensure correlation between LM images and EM section images. Ultrathin sections were collected and imaged as above.

Image quantification analysis

Cell composition:

For the cell type quantifications, z-stack projections with an equal number of z-stacks were used. 4 or more non-overlapping fields of each section were imaged using a 63x objective on a SPE microscope. Three different sections were quantified per mouse (≥4 animals per group). The area of each quantified field was 0.0135mm^2. Confocal images were counted manually using Fiji software. Within TEM images, each cell type was identified and quantified based on their morphology and the presence of a nucleus. mSCs, nmSCs, pericytes and endothelial cells are morphologically very different, however, we were unable to differentiate between the pericyte-like cells and macrophages. Therefore these cell types were quantified as a single category.

Determination of proliferation rates

Turnover of each cell-type was determined by using measurements taken following 30 days of continuous EdU administration. The calculation used was: proportion of each cell-type/(proportion of each proliferating cell-type x the total proliferation rate at 30 days).

ECM analysis:

For area measurements, 4 or more different fields of each section were imaged, with three sections counted for each mouse (≥4 mice per group). Images were converted to 8-bit grey scale TIFF images using Fiji software. Each image was thresholded and made binary. The thresholded area was outlined using the "Create Selection" function and the immunostained area quantified using the measurement function. For intensity measurements, projections used an equal number of z-stacks. The intensity of 9 different fields per image was measured and averaged using Fiji software (3 images were acquired for each section, 3 sections per animal, 6 animals per group).

Axon quantification:

The diameter of individual axons measured from 6 images per mouse and 8 mice per group was binned to assess distribution. All measurements were done with Photoshop to draw the axons and Fiji software was used to measure their diameter. Total number of myelinated axons were counted from 3 imaged sections of the entire nerve per mouse (n= 5 mice per group).

Development • Supplementary information

Table S1: Antibodies

Antibodies	Source	Identifier
Chicken polyclonal anti myelin protein zero (P0)	Abcam	Cat# ab39375 RRID: AB_881430
Rabbit polyclonal anti-nerve growth factor (NGF-receptor) p75	Millipore	Cat# AB1554 RRID: AB_90760
Rabbit polyclonal S100	DAKO	Cat# Z0311 RRID: AB_10013383
Rabbit polyclonal anti-Iba1	WAKO	Cat# 019-19741 RRID: AB_839504
Rat anti-mouse CD31 platelet endothelial cell adhesion molecule (PECAM)	BD Biosciences	Cat# 553370 RRID: AB_394816
Chicken polyclonal anti-200kD neurofilament	Abcam	Cat# ab4680 RRID: AB_30456
Rabbit polyclonal anti-laminin	Abcam	Cat# ab11575 RRID: AB_298179
Rabbit polyclonal anti-collagen III	Abcam	Cat# ab7778 RRID: AB_306066
Mouse anti-human fibronectin	Sigma-Aldrich	Cat# FN-3E2 RRID: AB_476981
Rabbit polyclonal anti-NG2 chondroitin sulfate proteoglycan antibody	Abcam	Cat# ab5320 RRID: AB_11213678
Rabbit monoclonal anti-PDGF Receptor Beta (Y92)	Abcam	Cat# ab 32570 RRID: AB_777165
Mouse monoclonal anti-actin alpha-smooth muscle- Cy3 conjugated (Clone 1A4)	Sigma-Aldrich	Cat# C6198 RRID: AB_476856
Chicken polyclonal anti-GFP	Abcam	Cat# ab13970 RRID: AB_300798
Rabbit polyclonal anti-glucose transporter 1 (GLUT1)	Abcam	Cat# ab652 RRID: AB_305540
Rat anti-mouse F4/80	Bio-Rad / AbD Serotec	Cat# MCA497G RRID: AB_872005
Rat monoclonal anti-NG2 chondroitin sulfate proteoglycan antibody	Thermo Fisher	Cat# MA5-24247 RRID:AB_2606388

Table S2: Transgenic mice

Mice strains	Reference	Identifier
Mouse: P0A-Cre (P0-Cre)		RRID: IMSR_RBRC01459
Mouse: PLP-eGFP	(Mallon et al., 2002)	N/A
Mouse: NG2-dsRedBAC (NG2-dsRed)	(Zhu et al., 2008)	RRID: IMSR_JAX:008241
Mouse: B6.Cg-*Gt(ROSA)26Sor*$^{tm9(CAG-tdTomato)Hze}$ (R26R-tdTomato)		RRID: IMSR_JAX:007909
Mouse: Tg(Mpz-cre/ERT2)2Ueli (P0-CreER^{T2})	(Leone et al., 2003)	RRID: IMSR_ MGI: 2663097
Mouse: R26R-YFP	(Srinivas et al., 2001)	
Mouse: Gt(ROSA)26Sor$^{tm1(CAG-Brainbow2.1)Cle}$ (R26R-Confetti)	(Snippert et al., 2010)	RRID: IMSR_JAX:013731
Mouse: P0-CreER^{T2}:R26R-YFP:Nf1$^{fl/fl}$	(Ribeiro et al., 2013)	

Development • Supplementary information

Table S3: Genotyping primers

**Genotyping primers**		
Primer for genotyping: P0-Cre 1: CGGTCGATGCAACGAGTGATGAG	This paper	N/A
Primer for genotyping: P0-Cre 2: CCAGAGACGGAAATCCATCGCTC	This paper	N/A
Primer for genotyping: NF1 Common CTT CAG ACT GAT TGT TGT ACC TGA		RRID: IMSR_JAX:017639
Primer for genotyping: NF1 Wild Type Reverse ACC TCT CTA GCC TCA GGA ATG A		RRID: IMSR_JAX:017639
Primer for genotyping: NF1 Mutant Reverse TGA TTC CCA CTT TCT GGT TCT AAG		RRID: IMSR_JAX:017639
Primer for genotyping: Confetti Mutant Forward GAA TTA ATT CCG GTA TAA CTT CG		RRID: IMSR_JAX:013731
Primer for genotyping: Confetti Wild Type Forward AAA GTC GCT CTG AGT TGT TA		RRID: IMSR_JAX:013731
Primer for genotyping: Confetti common CCA GAT GAC TAC CTA TCC TC		RRID: IMSR_JAX:013731
Primer for genotyping: R26R Mutant Reverse GCG AAG AGT TTG TCC TCA ACC-3	(Srinivas et al., 2001)	
Primer for genotyping: R26R Reverse GGA GCG GGA GAA ATG GAT ATG	(Srinivas et al., 2001)	
Primer for genotyping: R26R Common AAA GTC GCT CTG AGT TGT TAT	(Srinivas et al., 2001)	
Primer for genotyping: Tomato Wild Type Forward AAG GGA GCT GCA GTG GAG TA		RRID: IMSR_JAX:007909
Primer for genotyping: Tomato Wild Type Reverse CCG AAA ATC TGT GGG AAG TC		RRID: IMSR_JAX:007909
Primer for genotyping: Tomato Mutant Reverse GGC ATT AAA GCA GCG TAT CC		RRID: IMSR_JAX:007909
Primer for genotyping: Tomato Mutant Forward CTG TTC CTG TAC GGC ATG G		RRID: IMSR_JAX:007909
Primer for genotyping: Forward TTC CTT CGC CTT ACA AGT CC		RRID: IMSR_JAX:008241
Primer for genotyping: Reverse GAG CCG TAC TGG AAC TGG		RRID: IMSR_JAX:008241

Supplementary References

Leone, D. P., Genoud, S., Atanasoski, S., Grausenburger, R., Berger, P., Metzger, D., Macklin, W. B., Chambon, P. and Suter, U. (2003). Tamoxifen-inducible glia-specific Cre mice for somatic mutagenesis in oligodendrocytes and Schwann cells. *Molecular and cellular neurosciences* **22**, 430-440.

Mallon, B. S., Shick, H. E., Kidd, G. J. and Macklin, W. B. (2002). Proteolipid promoter activity distinguishes two populations of NG2-positive cells throughout neonatal cortical development. *The Journal of neuroscience : the official journal of the Society for Neuroscience* **22**, 876-885.

Ribeiro, S., Napoli, I., White, I. J., Parrinello, S., Flanagan, A. M., Suter, U., Parada, L. F. and Lloyd, A. C. (2013). Injury signals cooperate with Nf1 loss to relieve the tumor-suppressive environment of adult peripheral nerve. *Cell reports* **5**, 126-136.

Snippert, H. J., van der Flier, L. G., Sato, T., van Es, J. H., van den Born, M., Kroon-Veenboer, C., Barker, N., Klein, A. M., van Rheenen, J., Simons, B. D., et al. (2010). Intestinal crypt homeostasis results from neutral competition between symmetrically dividing Lgr5 stem cells. *Cell* **143**, 134-144.

Srinivas, S., Watanabe, T., Lin, C. S., William, C. M., Tanabe, Y., Jessell, T. M. and Costantini, F. (2001). Cre reporter strains produced by targeted insertion of EYFP and ECFP into the ROSA26 locus. *BMC developmental biology* **1**, 4.

Zhu, X., Bergles, D. E. and Nishiyama, A. (2008). NG2 cells generate both oligodendrocytes and gray matter astrocytes. *Development* **135**, 145-157.

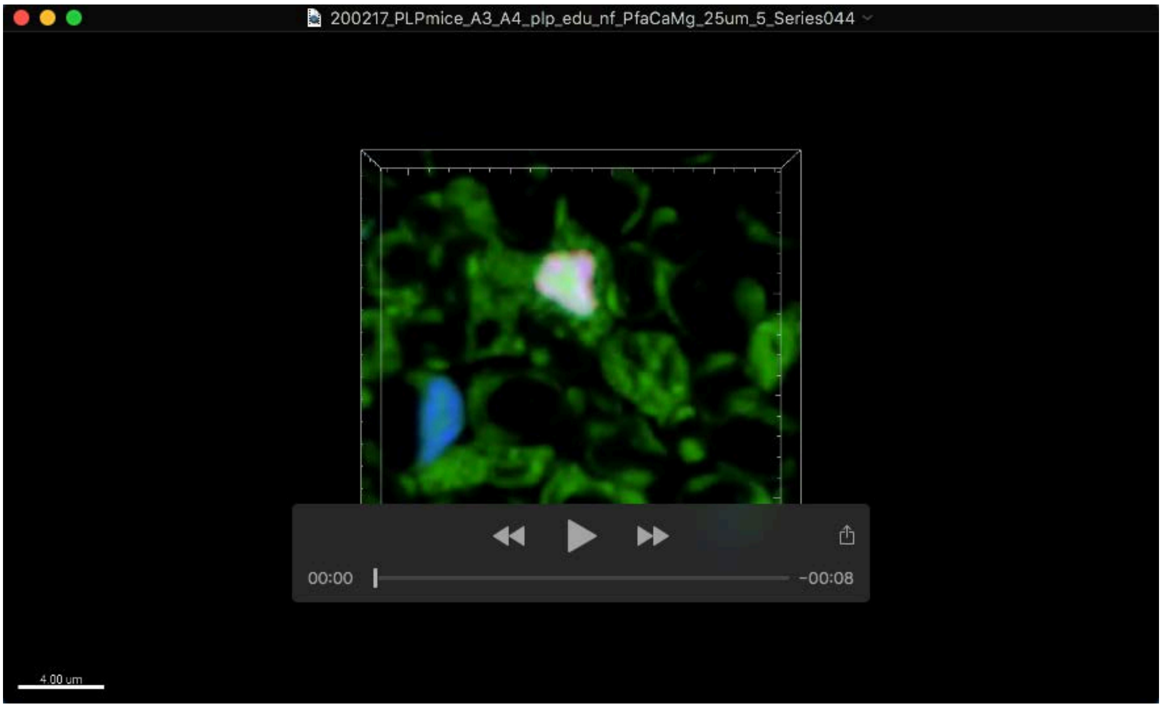

Movie 1: Non myelinating Schwann cells proliferate slowly in adult peripheral nerve.

Movie shows a rotating 3D projection of Z-stacks of confocal images of a transverse section ((20μm) of a sciatic nerve isolated from a PLP-eGFP mouse that had been treated with EdU continuously for 30 days prior to culling. The images were processed using Imaris software. The movie highlights a EdU+ (red), non-myelinating Schwann cell (eGFP+) associated with small calibre axons (neurofilament, white).
